# DMT1-dependent endosome-mitochondria interactions regulate mitochondrial iron translocation and metastatic outgrowth

**DOI:** 10.1038/s41388-023-02933-x

**Published:** 2024-01-06

**Authors:** Jonathan Barra, Isaiah Crosbourne, Cassandra L. Roberge, Ramon Bossardi-Ramos, Janine S. A. Warren, Kailie Matteson, Ling Wang, Frances Jourd’heuil, Sergey M. Borisov, Erin Bresnahan, Jose Javier Bravo-Cordero, Ruslan I. Dmitriev, David Jourd’heuil, Alejandro P. Adam, John M. Lamar, David T. Corr, Margarida M. Barroso

**Affiliations:** 1 Department of Molecular and Cellular Physiology, Albany Medical College, Albany, NY 12208 USA; 2 Department of Medicine, Division of Hematology and Oncology, Tisch Cancer Institute, Icahn School of Medicine at Mount Sinai, New York, NY 10029 USA; 3 Department of Biomedical Engineering, Binghamton University, Binghamton, NY 13902 USA; 4 Institute of Analytical Chemistry and Food Chemistry, Graz University of Technology Stremayrgasse 9, 8010 Graz, Austria; 5 Tissue Engineering and Biomaterials Group, Department of Human Structure and Repair, Faculty of Medical and Health Sciences, Ghent University, C. Heymanslaan 10, 9000 Ghent, Belgium; 6 Department of Biomedical Engineering, Rensselaer Polytechnic Institute, Troy, NY 12180-3590 USA

**Keywords:** Metastasis, Endosomes

## Abstract

Transient early endosome (EE)-mitochondria interactions can mediate mitochondrial iron translocation, but the associated mechanisms are still elusive. We showed that Divalent Metal Transporter 1 (DMT1) sustains mitochondrial iron translocation via EE-mitochondria interactions in triple-negative MDA-MB-231, but not in luminal A T47D breast cancer cells. DMT1 silencing increases labile iron pool (LIP) levels and activates PINK1/Parkin-dependent mitophagy in MDA-MB-231 cells. Mitochondrial bioenergetics and the iron-associated protein profile were altered by DMT1 silencing and rescued by DMT1 re-expression. Transcriptomic profiles upon DMT1 silencing are strikingly different between 2D and 3D culture conditions, suggesting that the environment context is crucial for the DMT1 knockout phenotype observed in MDA-MB-231 cells. Lastly, in vivo lung metastasis assay revealed that DMT1 silencing promoted the outgrowth of lung metastatic nodules in both human and murine models of triple-negative breast cancer cells. These findings reveal a DMT1‐dependent pathway connecting EE-mitochondria interactions to mitochondrial iron translocation and metastatic fitness of breast cancer cells.

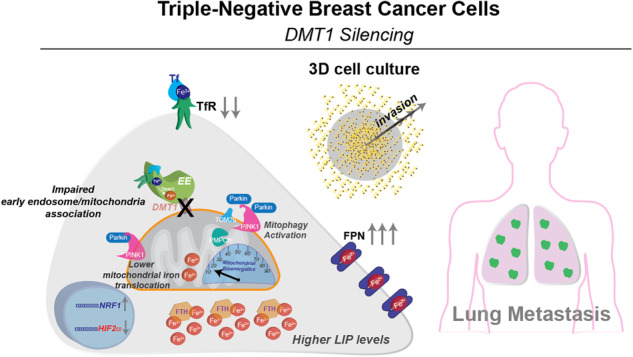

## Introduction

Iron is essential for the activity of key proteins and enzymes required for proper cellular functioning, and because of its low bioavailability, optimized mechanisms for iron absorption and conservation are required [1]. Importantly, there are no known excretory iron mechanisms in mammals, hence it accumulates inside the body over a lifetime [2]. Iron homeostasis is tightly regulated to limit intracellular labile iron pool (LIP), which is associated with the generation of reactive oxygen species (ROS) [3]. In non-cancerous cells, ROS increase is harmful but in cancer cells, iron increase and subsequent ROS overproduction can enhance their invasive and metastatic capacity by inducing loss of matrix attachment, promoting tumor angiogenesis, and increasing DNA damage [4].

Iron is associated with a higher risk for several cancer types [5] and recent studies show an association between the risk of breast cancer and iron levels [6, 7]. Regarding associated mechanisms, it has been suggested that in cancer cells, the accumulation of iron into the mitochondria, the main cellular iron sink, can rewire iron metabolism homeostasis toward adaptation to malignant features, including enhancement of growth and metastasis [8].

In circulation, ferric iron is mainly transported associated with transferrin (Tf) in the plasma [9]. Tf-bound ferric iron can be internalized by cells via transferrin receptor (TfR)-mediated endocytosis [10, 11]. Once internalized by peripheral cells, iron-bound Tf is readily sorted to endosomes, where iron is released from Tf upon exposure to the lower pH of the endosomal lumen. Then, ferric iron is reduced by the six-transmembrane epithelial antigen of the prostate 3 (Steap3) ferrireductase and translocated from early endosomes (EE) to mitochondria via transient “kiss-and-run” interactions. These inter-organelle contacts facilitate the direct translocation of iron into the mitochondria, bypassing the cytosol [12–14]. Alternatively, ferrous iron can be exported to the cytosol by the endosomal divalent metal transporter 1 (DMT1)-isoform II [15–17]. In the cytoplasm, iron can be either bound to specific chaperones for transport to distinct cellular compartments, stored in cytosolic ferritin (FTH), or exported via ferroportin (FPN), the only known mammalian cellular iron exporter [18]. Alternatively, as the main cellular iron sink, mitochondria can either store iron in mitochondrial ferritin or utilize it for heme and iron-sulfur clusters biosynthesis, which are components of important molecules such as hemoglobin and respiratory complexes I-III, respectively [19]. Hence, it is important to investigate how these various mechanisms could impact cancer cell malignancy.

DMT1 (or SLC11A2) is comprised of 12 transmembrane domains with *N*-glycosylation sites at C-and N-terminal tails, both facing the cytosol [20]. Besides iron, DMT1 can transport other divalent metals including Mn, Co, and Cu [20], although with less molecular affinity. Alternative splicing can generate two isoforms of DMT1 mRNA, DMT1-I and DMT1-II. After proper folding in the ER and Golgi apparatus, where they undergo N-linked glycosylation, each DMT1 isoform follows different sorting pathways. As mentioned, DMT1-isoform I is located mainly at the plasma membrane in the duodenal enterocytes [20–22], whereas, DMT1-isoform II is more abundant in non-epithelial cells where it is located at early/recycling endosomes [15–17, 23]. Structurally, the C-terminal domain of DMT1-II contains a retromer complex binding site, allowing it to localize to the early/recycling endosomal compartment [17, 24]. Interestingly, DMT1-II henceforth referred to as DMT1, has been localized at endosomes as well as at the outer mitochondrial membrane (OMM) [25], suggesting its involvement in mitochondrial iron translocation. Also, DMT1 has been identified as a major regulator of mitochondrial membrane potential [26]. Moreover, DMT1 overexpression has been shown to increase mitochondrial iron uptake [27]. However, the exact role of DMT1 in the mechanisms regulating EE-mitochondria interactions, mitochondrial iron translocation and mitochondrial function, as well as its role in cancer cell malignancy have been mainly unexplored.

In the present work, we first evaluated the role of endosomal DMT1 in transient “kiss-and-run” EE-mitochondria interactions, mitochondrial iron translocation, and cytoplasmic iron regulation in different cell lines representative of the breast cancer subtypes triple-negative (MDA-MB-231) and luminal A (T47D). We found that DMT1 can be present in EE as well as in association with the OMM, acting as a bridge between EE and mitochondria organelles. These DMT1-mediated bridging events are more frequent in MDA-MB-231 than in T47D breast cancer cells. In agreement, DMT1 also regulates mitochondrial iron translocation in MDA-MB-231 but not in T47D cells. Cytoplasmic iron levels, as well as mitochondrial ROS generation, increase upon DMT1 silencing in MDA-MB-231 breast cancer cells. Mitochondrial bioenergetics was severely impaired upon DMT1 silencing in MDA-MB-231 cells. Moreover, PINK1/Parkin-dependent mitophagy is regulated by DMT1 via the association of PINK1 at the OMM with the DMT1 interactor PMPCB, a peptidase regulating PINK1 turnover, in MDA-MB-231 cells. Interestingly, transcriptome analysis revealed significant differences upon DMT1 ablation between 2D and 3D cell culture conditions. Furthermore, we observed that DMT1 silencing induced lower invasive migration in 2D cell culture conditions but conversely increased MDA-MB-231 invasive capacity in 3D cell culture (spheroids). Concurringly, in vivo, DMT1 silencing in human and murine models of triple-negative breast cancer increases the metastatic outgrowth of MDA-MB-231 cells upon lung metastatic colonization. Immunofluorescence analysis of lung metastases shows that DMT1 regulates the EE-mitochondria inter-organelle association similarly to 2D cell culture conditions. Furthermore, analyzes of triple-negative MDA-MB-231 derived from metastases in vivo showed increases in LIP and significant delay in mitochondrial iron translocation compared to parental cells, like DMT1 KO cells. These findings reveal a DMT1-dependent pathway connecting EE-mitochondria interactions to mitochondrial iron translocation, mitochondrial metabolism, and metastatic fitness of triple-negative breast cancer cells.

## Results

### DMT1 bridges EE and mitochondria in triple-negative MDA-MB-231 but not in luminal A T47D breast cancer cells

**Fig. 1:**
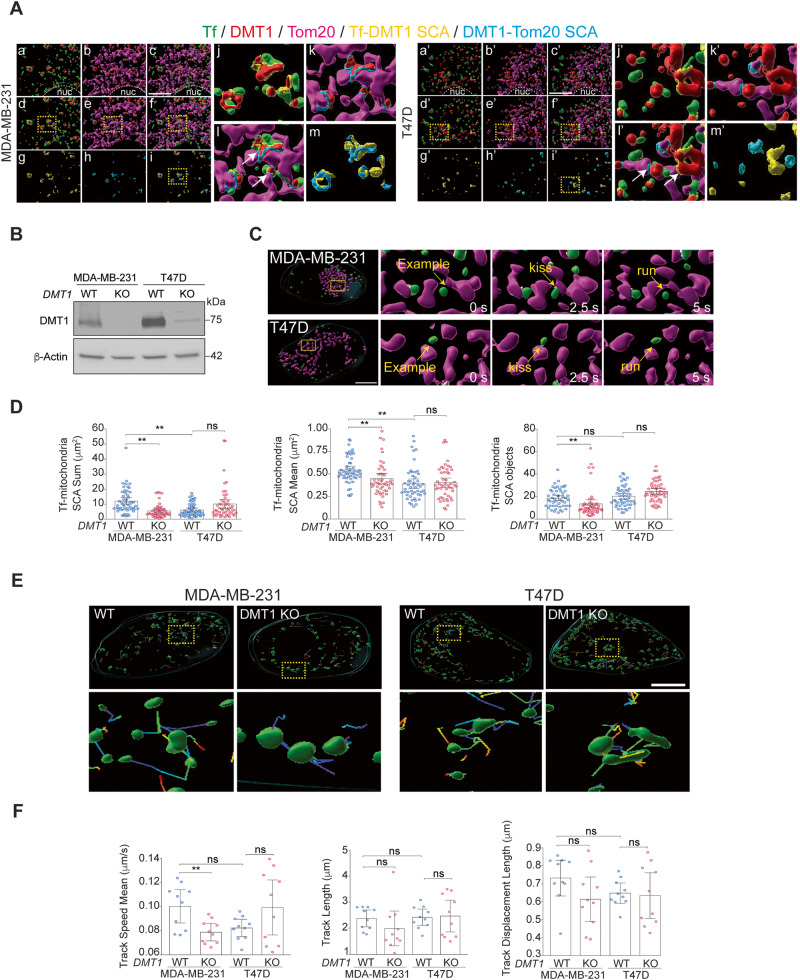
DMT1 bridges EE and mitochondria and regulates their inter-organelle contacts in MDA-MB-231 but not in T47D breast cancer cells. **A** MDA-MB-231 (a–m) and T47D (a’–m’) cells were subjected to fluorescently labeled-Tf uptake (green) via a short pulse-chase protocol to load the early endosomes followed by immunofluorescence analysis of DMT1 (red), and Tom20 (magenta). Fluorescent z-stacks images were deconvoluted using the integrated small volume computational clearing (SVCC) algorithm in the Leica Thunder microscope and then 3D rendered using Imaris software 9.6 (Bitplane). Inter-organelle contacts between Tf-containing EE-DMT1 (yellow) and DMT1-Tom20 (light blue) were calculated using the “Surface Contact Area” (SCA) Imaris XTension integrated plugin. Representative images are shown for Tf and DMT1 (a, a’); DMT1 and Tom20 (b, b’); Tf, DMT1, and Tom20 (c, c’); Tf, DMT1 and Tf-DMT1 SCA (d, d’); DMT1, Tom20 and DMT1-Tom20 SCA (e, e’); Tf, DMT1, Tom20, Tf-DMT1 SCA and DMT1-Tom20 SCA (f, f’); Tf-DMT1 SCA (g, g’); DMT1-Tom20 SCA (h, h’); Tf-DMT1 SCA and DMT1-Tom20 SCA (i, i’). Regions-of-interest indicated by dotted squares shown in (d–f & i; d’–f’ & i’) are shown upon magnification in (j–m and j’–m’), respectively. A lower association between EE-DMT1 and Tom20-DMT1 SCAs in T47D vs. MDA-MB-231 is observed. Nuc = nucleus; Scale bar = 10 μm. **B** Immunoblot shows CRISPR/Cas9 mediated DMT1 silencing in indicated cell lines. β-actin immunoblot is shown as a loading control. **C** MDA-MB-231 (top) and T47D (bottom) cells were subjected to fluorescently labeled-Tf uptake (green) via a short pulse-chase protocol to load the early endosomes followed by time-lapse live cell imaging using Thunder microscopy with SVCC deconvolution algorithm. Examples of 3D reconstructions of fluorescently labeled Tf-containing EE (green), Mitotracker-labeled mitochondria (magenta), and Tf-containing EE-mitochondria SCA (white) from time-lapse live-cell imaging experiments (interval 2.5–3 s, 4 frames per second, total time 45 s) show examples of “kiss-and-run” EE-mitochondria interactions (arrows) in MDA-MB-231 and T47D cells. Images were analyzed using Imaris 9.6 software. Scale bar = 10 μm. **D** Tf-containing EE-mitochondria SCA sum (**left panel**), mean (**middle panel**), and the number of objects (**right panel**) in MDA-MB-231 and T47D WT and DMT1 KO are shown. A significant decrease in the total amount of EE-mitochondria SCA sum and mean per cell is observed upon DMT1 silencing in MDA-MB-231 but not in T47D. Parameters were calculated using data from 10 individual cells in 5 consecutive time intervals per condition and analyzed separately (*n* = 50). One-way ANOVA with Bonferroni post-hoc test. **p < 0.01. ns: non-significant (*p* > 0.05). **E** Top row shows representative images displaying Tf-containing EE and their respective endosomal tracks obtained from live cell imaging experiments described above. Yellow dotted squares indicate the corresponding magnified regions shown in the bottom row. **F** Fluorescent z-stack images were 3D rendered and analyzed using IMARIS 9.6 software to quantify endosomal track speed mean, track length, and track displacement length per cell (*n* = 10 cells). One-way ANOVA with Bonferroni post-hoc test. **p < 0.01. ns: non-significant (*p* > 0.05). Scale bar = 10 μm.

Here, we studied the role of DMT1 in endosome-mitochondria interactions in breast cancer cells. First, two cell lines representative of two different types of breast cancer, MDA-MB-231 (triple negative) and T47D (Luminal A) were incubated for 2 min with fluorescently labeled Tf (green), chased for 2–5 min to label EE, and then subjected to immunofluorescence against DMT1 (red), and Tom20 (magenta), a marker of the OMM. After 3D rendering, we analyzed inter-organelle contacts via the Surface Contact Area (SCA) Imaris XTension applied to either Tf with DMT1 (yellow) and DMT1 with Tom20 (light blue) (Fig. 1A). Using this pulse-chase protocol, allowed for Tf to localize to early endosomal compartments in these breast cancer cell lines, as shown previously [28]. Here, we showed that in both MDA-MB-231 and T47D cells, DMT1 co-distributed with Tf as well as with Tom20, which is in agreement with previous reports showing co-distribution of DMT1 with either EE or mitochondria [17, 24, 27]. DMT1 fluorescence intensity was higher in the EE but lower in the OMM in MDA-MB-231 compared to T47D cells (Fig. S1A), suggesting less association of DMT1 with EE but an increased association of DMT1 with OMM in T47D cells.

Interestingly, in MDA-MB-231 cells, almost all the Tf-DMT1 SCA events were observed in proximity to DMT1-Tom20 SCA events **(**Fig. 1A, j–m**)**. In contrast, in T47D cells, the SCAs of Tf-DMT1 and DMT1-Tom20 were found mainly separated (Fig. 1A, j–m). Taken together, these data indicate that DMT1 is found associated with Tf-containing EE as well as with mitochondria in breast cancer cells. However, the presence of DMT1 acting as a bridge between Tf-containing EE and mitochondria is more frequently observed in triple-negative MDA-MB-231 than in luminal A T47D cells.

### DMT1 silencing decreases EE-mitochondria association in MDA-MB-231 but not in T47D breast cancer cells

In non-cancerous epithelial cells, we previously reported that the kinetics of direct and transient “kiss-and-run” Tf-containing EE-mitochondria interactions are modulated by the availability of intra-endosomal iron [12]. However, the mechanisms and molecules involved in this process, and how they can be regulated in cancer cells, are mainly unexplored. Since DMT1 is detected bridging Tf-containing EE with mitochondria in MDA-MB-231 breast cancer cells, we then evaluated the role of DMT1 in EE-mitochondria association by silencing DMT1 using CRISPR/Cas9 in MDA-MB-231 or T47D breast cancer cell lines (Fig. 1B). Cells were co-labeled with a 2 min pulse of fluorescently labeled Tf and Mitotracker to study the effect of DMT1 knock-out (KO) on Tf-containing EE-mitochondria “kiss-and-run” transient interactions. Time-lapse live-cell imaging was performed, and Z-stacks were acquired using the DMi8 Thunder microscope (Leica) with an integrated computational clearing algorithm to visualize the “kiss and run” inter-organelle contacts between Tf-containing EE and mitochondria (Fig. 1C). After 3D rendering, we measured the sum, mean, and number of the SCA between both organelles as an indicator of the total Tf-containing EE-mitochondria interaction per cell (Fig. 1D). The sum and mean of Tf-containing EE-mitochondria SCAs were higher in the MDA-MB-231 WT cells than in the T47D WT cells (Fig. 1D). Upon DMT1 ablation, the sum, mean, and total number of Tf-containing EE-mitochondria SCAs per cell decreased significantly in MDA-MB-231, but not in T47D cells (Fig. 1D). Consistently, we observed a decreasing trend in the duration of EE-mitochondria interaction upon DMT1 ablation in MDA-MB-231 cells (Fig. S1B). Using immunofluorescence on fixed MDA-MB-231 cells we corroborated that the SCA sum between EEA1, an EE marker, and Tom20 significantly decreases upon DMT1 silencing, although the SCA mean was similar between WT and DMT1 KO cells (Fig. S1C). Consistently with DMT1 acting as a bridge between endosomes and mitochondria more frequently in MDA-MB-231 than in T47D cells, MDA-MB-231 cells show a significant decrease in EE-mitochondria interactions upon ablation of DMT1. These results indicate a role for DMT1 in the establishment of inter-organelle EE-mitochondria contacts only in MDA-MB-231.

### DMT1 silencing alters organelle morphology and decreases EE speed in MDA-MB-231 cells

Importantly, in agreement with our previous findings [28], we found a lower mean value of the EE area in T47D WT, compared to MDA-MB-231 WT cells (Fig. S1D). An increase in Tf-containing EE area, upon DMT1 ablation in T47D, but not in MDA-MB-231 cells, suggests a role for DMT1 in the regulation of EE morphology only in T47D. Moreover, we observed a significant decrease in both Tf-containing EE area sum and number upon DMT1 ablation in MDA-MB-231, but not in T47D cells (Fig. S1D). Upon 3D rendering of mitochondrial objects, we showed that DMT1 silencing also decreases mitochondrial area sum in MDA-MB-231, but not in T47D cells. However, the mitochondria area mean was increased in both cell lines (Fig. S1E). The number of mitochondrial objects was reduced upon DMT1 ablation in MDA-MB-231 but not in T47D cells (Fig. S1E). These results suggest that DMT1 loss might lead to reduced Tf-containing EE-mitochondria contacts via decreased total EE and mitochondria compartments. Nevertheless, DMT1 loss increased mitochondrial tubulation and reduced fragmentation in MDA-MB-231 cells. In contrast, loss of DMT1 in T47D cells led to increased mitochondrial surface, but not to a reduced number of objects. Overall, these data showed that silencing of DMT1 alters the morphology of EE and mitochondria organelles in both cell lines but decreases EE-mitochondria interaction only in MDA-MB-231.

Previously, we demonstrated that the blocking of iron release from Tf at the EE lumen can regulate both EE speed and duration of the EE-mitochondria transient interactions [12]. Interestingly, we found that DMT1 silencing decreases EE speed in MDA-MB-231, but not in T47D breast cancer cells (Fig. 1E, F**)**. Frequency distribution analysis of EE speed showed lower EE speeds upon DMT1 ablation only in MDA-MB-231 **(**Fig. S1F). A higher endosomal speed in the non-interacting EE in both DMT1 WT and DMT1 KO cells was also observed in MDA-MB-231 cells (Fig. S1B). Lower EE speed can also play a role in the reduction of both the number and the sum of EE-mitochondria association upon DMT1 silencing in MDA-MB-231 cells. Taken together these results indicate that DMT1 plays an important role in EE-mitochondria interactions and endosomal dynamics in MDA-MB-231 but not T47D cells.

### DMT1 is required for mitochondrial iron translocation in MDA-MB-231 but not in T47D breast cancer cells

**Fig. 2:**
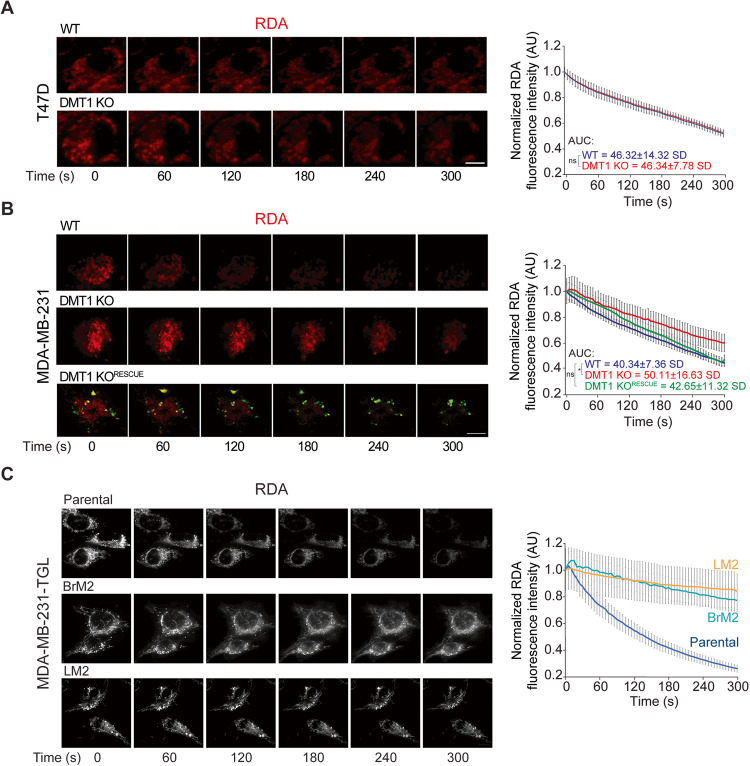
DMT1 regulates mitochondrial iron translocation in MDA-MB-231 cells. **A** T47D and **B** MDA-MB-231 cells were incubated with RDA (a biosensor that localizes to mitochondria and undergoes rapid quenching upon mitochondrial iron translocation) for 15 min and then subjected to live cell imaging. Fluorescence images (**left**) and normalized fluorescence quantification graphs (**right**) show RDA dequenching levels from 0 to 300 s (s). RDA fluorescence intensity decay was analyzed using ImageJ in 20 cells per condition. Unpaired *t*-test and ANOVA with Bonferroni post-hoc test were used for statistical analysis of the area under the curve (AUC) in each condition (*p < 0.05). In MDA-MB-231, but not in T47D, DMT1 ablation induces a significant delay in RDA dequenching indicating lower mitochondrial iron translocation. DMT1-GFP re-expression in DMT1 KO cells (DMT1 KO^RESCUE^) significantly rescued the decrease in mitochondrial iron translocation in DMT1 KO cells. Scale bar = 10 μm. **C** Parental MDA-MB-231-TGL cells and its metastatic derived clones (Brain, BrM2 and Lung, LM2) were incubated with RDA for 15 min and then subjected to time-lapse live cell imaging using Thunder microscopy with integrated SVCC algorithm. Fluorescent images (**left**) and normalized fluorescence quantification graph (**right**) show RDA dequenching levels from 0 to 300 s. Metastatic-derived cell lines MDA-MB-231-TGL BrM2 and LM2 RDA-fluorescence intensity decay was analyzed using ImageJ software in 20 cells per condition. Scale bar = 10 μm.

Previously, we have shown that transient and direct interactions between EE and mitochondria can facilitate mitochondrial iron translocation in epithelial cells [12]. Here, we evaluated the involvement of DMT1 on mitochondrial iron translocation in both T47D and MDA-MB-231 breast cancer cells using the mitochondrial iron sensor probe RDA, which is rapidly quenched upon iron translocation into the mitochondria [12, 29]. Interestingly, in T47D cells DMT1 silencing did not alter RDA fluorescence quenching compared to T47D WT parental cells (Fig. 2A), indicating that in this cell line, DMT1 is not critical for mitochondrial iron translocation. However, in MDA-MB-231 cells, DMT1 ablation led to a delay in RDA fluorescence quenching compared to WT cells, suggesting a significant reduction of mitochondrial iron translocation (Fig. 2B). Re-expression of mouse-DMT1-GFP in MDA-MB-231 DMT1 KO significantly rescued mitochondrial iron translocation (Fig. 2B and Fig. S2A), indicating a specific role for DMT1 in the regulation of this process in MDA-MB-231 cells. Consistent with the results above, we found that the re-expressed mouse DMT1-GFP co-localized with EE in MDA-MB-231 cells (Fig. S2B). In MDA-MB-231 cells, but not in T47D cells, DMT1 silencing leads to reduced levels of chelatable intra-mitochondrial iron.

To further evaluate our observations in breast metastatic conditions, we used MDA-MB-231 cells (TGL parental) derived from lung (LM2), and brain (BrM2) metastases (kindly provided by Dr. Joan Massague) to evaluate mitochondrial iron translocation in these cell lines. Strikingly, we observed that mitochondrial iron translocation was dramatically decreased, in both metastasis-derived cell lines compared to parental MDA-MB-231 TGL cells (Fig. 2C). Overall, our results indicate that DMT1 regulates mitochondrial iron translocation in triple-negative MDA-MB-231 cells but not in luminal A T47D cells, raising the intriguing possibility of a differential requirement for DMT1 in the maintenance of adequate mitochondrial iron translocation across different breast cancer cell lines. Moreover, reduced iron mitochondrial translocation is detected in metastasis-derived triple-negative breast cancer cell lines, indicating that decreased accumulation of iron in mitochondria may be associated with metastatic processes.

### DMT1 regulates the labile iron pool (LIP) in triple-negative breast cancer cells

**Fig. 3:**
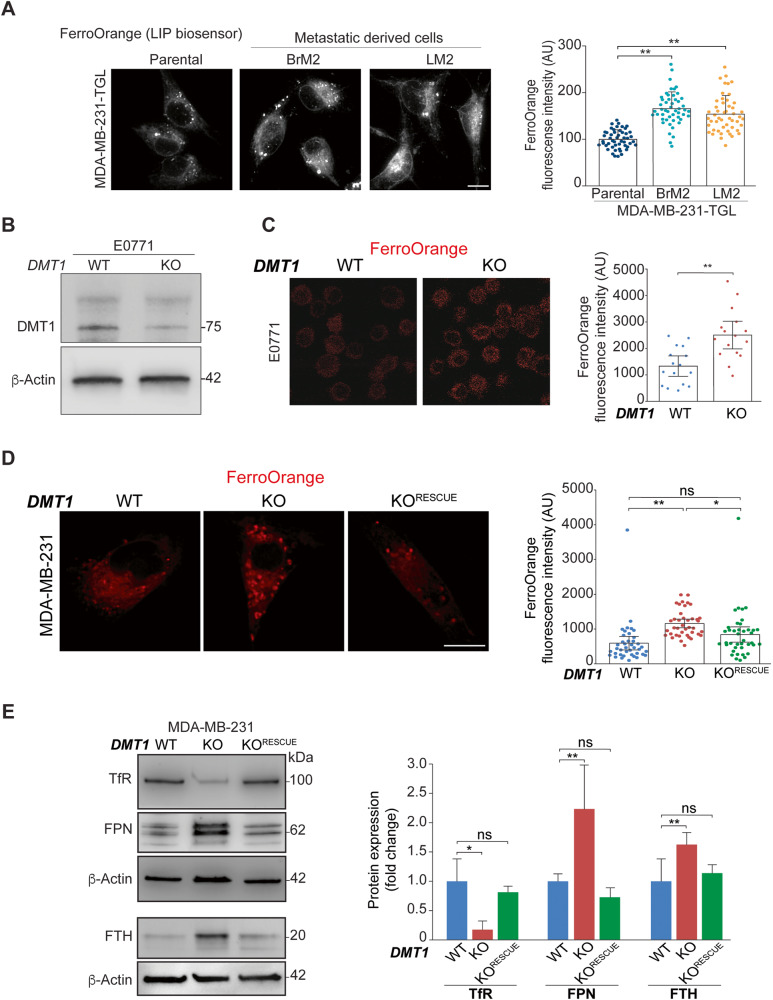
DMT1 regulates LIP and the expression levels of iron transport-related proteins in triple-negative breast cancer cells. **A** Fluorescent images (**left**) and normalized fluorescence quantification graphs (**right**) of LIP biosensor FerroOrange dye in metastatic derived cells (Brain, **BrM2** and Lung, **LM2**) and parental MDA-MB-231-TGL cells. The graph shows the quantification of fluorescence intensity in arbitrary units (AU). Metastatic-derived cell lines MDA-MB-231-TGL BrM2 and LM2 show significant increases in LIP levels compared to parental cells. FerroOrange fluorescence intensity was analyzed using ImageJ software in 40 cells per condition. One-way ANOVA with Bonferroni post-hoc test. **p < 0.01. ns: non-significant. Scale bar = 10 μm. **B** Immunoblot showing DMT1 silencing in mouse triple-negative breast cancer cell line E0771-GFP. β-Actin was used as a loading control. **C** Fluorescent images (**left**) and normalized fluorescence quantification graph (**right**) of LIP biosensor FerroOrange dye in E0771-GFP cells. The graph shows the quantification of fluorescence intensity in arbitrary units (AU). FerroOrange fluorescence intensity was analyzed using ImageJ software in 15 cells per condition. Unpaired *t*-test; **p < 0.01. Scale bar = 10 μm. **D** Fluorescent images (**left**) and normalized fluorescence quantification graphs (**right**) of LIP biosensor FerroOrange dye in MDA-MB-231 cells. The graph shows the quantification of fluorescence intensity in arbitrary units (AU). DMT1-GFP re-expression in DMT1 KO cells rescued the LIP increase induced by DMT1 silencing. FerroOrange fluorescence intensity was analyzed using ImageJ software in 40 cells per condition. One-way ANOVA with Bonferroni post-hoc test. *p < 0.05; **p < 0.01. ns: non-significant. Scale bar = 10 μm. **E** Immunoblot (**left**) and normalized densitometry quantification graphs (**right**) (*n* = 3) showed expression levels of TfR, FPN, and FTH in MDA-MB-231 WT, DMT1 KO, and DMT1 KO^RESCUE^ cells. β-Actin was used as a loading control. One-way ANOVA with Bonferroni post-hoc test. *p < 0.05; **p < 0.01. ns: non-significant.

Since mitochondrial iron translocation was shown to be decreased in metastatic triple-negative breast cancer cell lines, we tested whether LIP levels were elevated, as expected considering that iron accumulation in mitochondria is reduced. To evaluate LIP, we used the ferrous iron-specific fluorescent dye FerroOrange, which reacts with LIP in the cytoplasm. LIP describes the chemically reactive, kinetically exchangeable pool of intracellular nonprotein-bound iron (ferrous iron) that can either generate ROS via the Fenton reaction or be used as the raw material for iron cofactor synthesis, assembly, and insertion [2, 30]. Strikingly, we observed that LIP levels were significantly increased in both metastasis-derived cell lines compared to parental MDA-MB-231 TGL cells (Fig. 3A). Consistently with the ability of FerroOrange to react with ferrous iron throughout the cell, higher FerroOrange staining can be detected diffusively in the cytoplasm as well as in punctate-like structures. Therefore, the accumulation of labile iron may occur in the cytoplasm and membrane-bound punctate organelles, such as EE, late endosomes, or lysosomes, for example. Then, we tested whether DMT1 ablation in triple-negative breast cancer cells can also lead to increased LIP levels using FerroOrange. We used CRISPR/Cas9 to silence DMT1 in E0771, a well-characterized murine cellular model of triple-negative breast cancer [31] (Fig. 3B). As shown in Fig. 3C, DMT1 silencing increased the LIP levels in E0771 cells. In agreement, DMT1 ablation in MDA-MB-231 cells significantly increased LIP levels, an effect that was rescued by DMT1 re-expression in DMT1 KO cells in a statistically significant manner (Fig. 3D).

As mentioned above, the main cellular regulators of iron import, export, and storage are TfR, FPN, and FTH, respectively [1]. DMT1 silencing decreased TfR expression but increased FPN and FTH expression in MDA-MB-231 cells. All these effects were significantly rescued by DMT1 re-expression in DMT1 KO cells (Fig. 3E). Previously, it was shown that mitochondrial iron chelation induced by specific mitochondrial-targeted deferoxamine, increases mitochondrial superoxide levels [32]. Mitochondria are the main source of ROS and therefore mitochondrial ROS production and availability are tightly controlled in cells [33]. Here, we used the probe MitoSOX, which is readily oxidized by superoxide but not by other ROS- or RNS-generating systems [34], to selectively measure mitochondrial superoxide production. We observed a significant increase in mitochondrial superoxide levels upon DMT1 silencing in MDA-MB-231 cells (Fig. S2C). However, the fluorescence intensity of TMRM, which is preferred for its reduced mitochondrial binding and electron transport chain inhibition [35] did not change upon DMT1 ablation, thereby indicating that mitochondrial membrane potential is not significantly affected by DMT1 silencing in MDA-MB-231 cells (Fig. S2D). These results indicate that DMT1 silencing correlates with a significant disruption in the intracellular iron metabolism of triple-negative breast cancer cells. Interestingly, the expression level of iron transport regulators, such as TfR and FPN, is altered in a way consistent with an iron-depleted phenotype displaying decreased TfR and elevated FPN and FTH protein expression levels. However, we also detected a decrease in mitochondrial iron translocation, as well as an increase in LIP levels and mitochondrial ROS. These results suggest that DMT1 silencing can induce a novel and distinct iron-altered phenotype in triple-negative breast cancer cells.

### DMT1 silencing decreases mitochondrial oxygen consumption and glycolysis and activates mitophagy in MDA-MB-231 cells

**Fig. 4:**
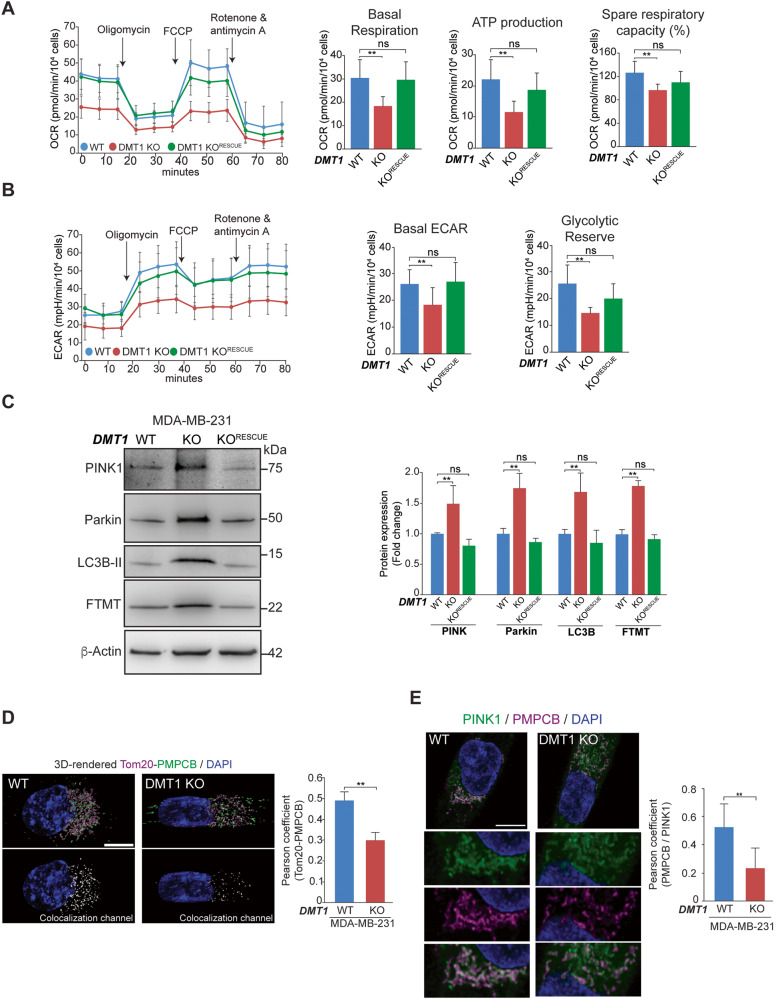
DMT1 silencing impairs oxidative/glycolytic mitochondrial metabolism and activates PINK1/Parkin-dependent mitophagy in MDA-MB-231 cells. **A, B** Oxygen consumption rate (OCR) and extracellular acidification rate (ECAR) in response to sequential treatment with oligomycin, FCCP, and Rotenone & Antimycin A of MDA-MB-231 WT, DMT1 KO, and DMT1 KO^RESCUE^ cells are shown. Data were normalized for the number of cells (40,000 cells/well) that were stained using Hoechst at the end of the assay (*n* = 3, 6–10 replicates per condition). Basal respiration, ATP production, and spare respiratory capacity were calculated using OCR data. Basal ECAR and glycolytic reserve were calculated using ECAR data. Bar charts: one-way ANOVA with Bonferroni post-hoc test. *p < 0.05; **p < 0.01. ns: non-significant. Scale bar = 100 μm. **C** Representative immunoblots (**left**) and normalized densitometry quantification graph (**right**) of PINK1, Parkin, LC3B-II, and mitochondrial ferritin (FTMT) in MDA-MB-231 WT, DMT1 KO, and DMT1 KO^RESCUE^ cells. β-Actin was used as a loading control. Bar charts: one-way ANOVA with Bonferroni post-hoc test (*n* = 3). *p < 0.05; **p < 0.01. ns: non-significant. **D** Immunofluorescence of Tom20 and PMPCB shows a decrease in colocalization between both proteins upon DMT1 silencing in MDA-MB-231. Pearson colocalization coefficient was analyzed using IMARIS 9.6 software in 25 cells per condition. Unpaired *t*-test. **p < 0.01. Scale bar = 10 μm. **E** Immunofluorescence of PINK1 and PMPCB shows a decrease in colocalization between both proteins, and mitochondrial fragmentation upon DMT1 silencing in MDA-MB-231. Pearson colocalization coefficient was analyzed using IMARIS 9.6 software in 25 cells per condition. Unpaired *t*-test. **p < 0.01. Scale bar = 10 μm.

Since iron is required for mitochondrial respiration and overall metabolism [36], we further analyzed the impact of this DMT1 silencing-mediated novel iron phenotype on mitochondrial bioenergetics, we used the Seahorse Mito Stress Test assay to simultaneously evaluate the oxygen consumption rate (OCR) and extra-cellular acidification rate (ECAR) in live cells. We found that loss of DMT1 led to a decrease in both mitochondrial respiration (basal OCR) and aerobic glycolysis (basal ECAR). Importantly, both parameters were restored to similar levels to WT cells following the reintroduction of DMT1 into MDA-MB-231 DMT1 KO cells (Fig. 4A, B). Moreover, basal respiration, ATP production, spare respiratory capacity, basal ECAR, and glycolytic reserve decreased upon DMT1 silencing and returned to basal levels upon DMT1 rescue in KO cells (Fig. 4A, B). Thus, consistently with its role in mitochondrial iron translocation, DMT1 is required for homeostatic mitochondrial respiration and glycolysis in MDA-MB-231 breast cancer cells.

Mitophagy is the cellular mitochondrial quality control process by which depolarized and/or damaged mitochondria are selectively subjected to degradation. Interestingly, recent studies have demonstrated that iron chelation induces mitophagy with deleterious effects on cancer cells, including breast cancer [32, 37]. Since DMT1 silencing results in reduced iron accumulation in mitochondria, we have analyzed the effect of DMT1 ablation in mitophagy. However, there is considerable controversy regarding whether mitophagy plays a suppressor or oncogenic role in tumor progression; such a role is likely dependent on the type of tumor evaluated [32, 37–39]. Here, we investigated the impact of DMT1 silencing on PINK1/Parkin-dependent mitophagy, as well as on FTMT expression, whose induction had been recently associated with iron chelation-induced mitophagy in cancer cells [37], and on the global autophagy marker LC3B-II in both MDA-MB-231 and T47D breast cancer cells. Strikingly, in T47D cells none of these markers were altered upon DMT1 silencing (Figure S2E) likely because, in T47D, DMT1 silencing does not affect mitochondrial iron translocation (Fig. 2A, B). In contrast, DMT1 ablation in MDA-MB-231 cells significantly induced PINK1/Parkin-dependent mitophagy, as well as FTMT and LC3B-II expression levels (Fig. 4C). Recovery towards basal levels of all these markers was achieved by re-expression of DMT1 in MDA-MB-231 DMT1 KO cells (Fig. 4C).

To explore the mechanism of DMT1-dependent mitophagy, we focused on the peptidase PMPCB, a recently described interactor of DMT1 [40]. PMPCB and PMPCA can establish the mitochondrial processing peptidase complex [41]. PINK1 is constitutively imported into the mitochondria, where it’s proteolytically degraded in a PMPCB-dependent manner [42]. Failure to import or to degrade PINK1 can lead to its accumulation on the OMM, hyperactivating PINK1/Parkin-dependent mitophagy [43]. Using immunofluorescence and subsequent Pearson coefficient colocalization analysis as previously described [28], we observed a significant decrease in the association between PMPCB and the OMM marker Tom20 upon DMT1 silencing in MDA-MB-231 cells (Fig. 4D). Moreover, we observed a decrease in the colocalization of PINK1 with PMPCB in MDA-MB-231 DMT1 KO cells (Fig. 4E), which indicates a deficiency in the recruitment of PMPCB towards the OMM for mitophagy prevention. Overall, these data show that DMT1 is involved in the crosstalk between PMPCB and OMM, and subsequent turnover of proteins, such as PINK1, thus, preventing mitophagy activation. Our results reveal a differential dependence of DMT1 in MDA-MB-231 and T47D for PINK1/Parkin-dependent mitophagy, which is likely connected to distinct regulation of mitochondrial iron homeostasis across different breast cancer cell lines.

### DMT1 silencing disrupts the aggregation of 3D multicellular tumoral constructs

**Fig. 5:**
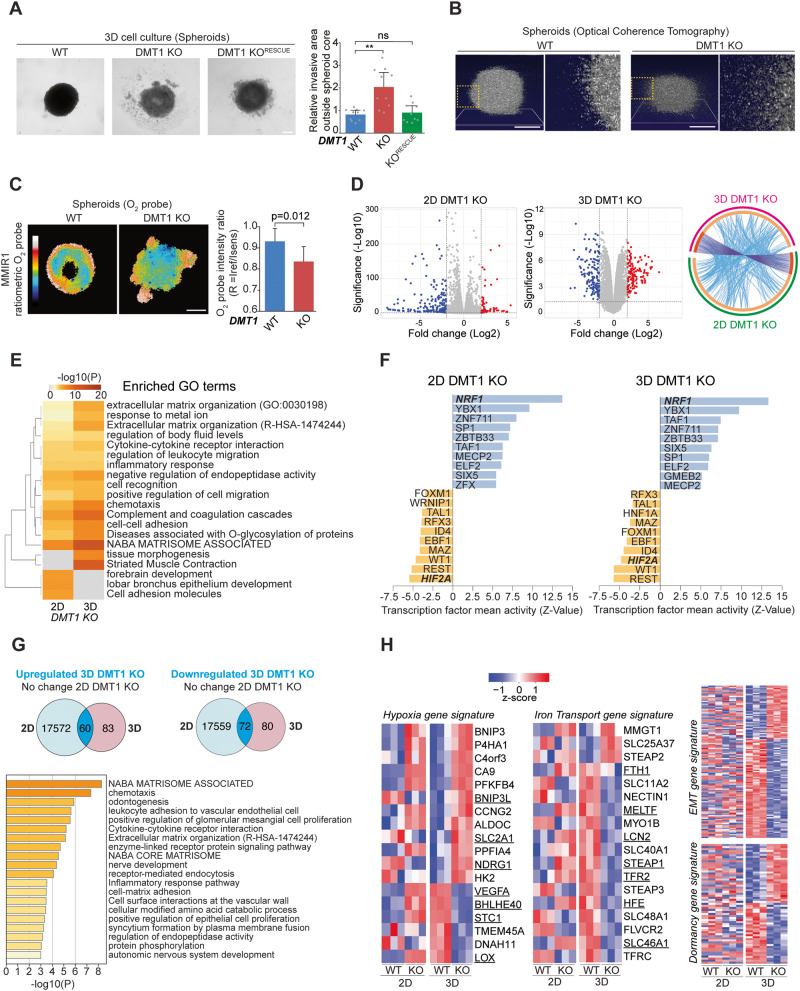
Functional and transcriptional effects of DMT1 silencing on 3D cell culture conditions. **A** Phase contrast images of 3D cell culture spheroids of MDA-MB-231 cells. Relative cellularity outside the spheroid core was quantified in WT, DMT1 KO, and DMT1 KO^RESCUE^ cells. Bar chart: one-way ANOVA with Bonferroni post-hoc test (10 spheroids per condition). *p < 0.05; **p < 0.01. ns: non-significant. Scale bar = 200 μm. **B** Isometric Optical Coherence Tomography (OCT) showing representative images of MDA-MB-231 WT and DMT1 KO spheroids at day 4 in 3D cell culture. Yellow dotted squares indicate the corresponding magnified regions showing higher cell dispersion at the periphery of DMT1 KO spheroids. Scale bar = 200 μm. **C** Intensity ratio images of MMIR1 probe in WT and DMT1 KO spheroids. The coded color bar represents the image’s MMIR1 O_2_ probe intensity ratio (R = Iref / Isens) distribution. The bar graph shows the relative oxygenation levels in WT and DMT1 KO spheroids. Unpaired *t*-test (6 spheroids per condition). Scale bar = 200 μm. **D** Volcano Plot shows differentially regulated genes upon DMT1 silencing in 2D and 3D cell culture conditions obtained from total RNA sequencing (padj value < 0.05 and fold change log2 > 2). Circos plot showing the overlap by genes and GO terms from 2D DMT1 KO vs. 3D DMT1 KO-differentially regulated genes. Outside arcs indicate the identity of each differentially expressed gene list (green: 2D DMT1 KO; magenta: 3D DMT1 KO). Inside arcs indicate the complete differentially expressed gene list of each condition. The dark orange section indicates the genes that appear in both lists and the light orange section indicates genes that are not shared between gene lists. Purple lines link the same genes that are shared in both gene lists. Blue lines link the genes sharing the same GO term. **E** Gene set enrichment analysis (Biological Processes Gene Ontology) using significant upregulated and downregulated genes. Heatmap showing top positively and negatively enriched gene sets ranked by FDR *q*-values. **F** ISMARA analysis shows a specific increase (purple) and decrease (green) in transcription factor activity (*Z*-value). **G** Venn diagrams showing the overlap between upregulated and downregulated genes versus no-change in 2D genes upon DMT1 KO in 3D cell culture conditions. Gene Ontology analysis (Biological Processes) of upregulated and downregulated genes upon DMT1 KO in 3D culture conditions. **H** Heatmap of gene expression values for significantly downregulated and upregulated genes for either breast cancer hypoxia, iron transport, EMT, and dormancy gene signatures upon DMT1 silencing in 2D and 3D cell culture conditions.

Although 2D cell cultures have been an invaluable resource for cancer cell biology studies, they cannot appropriately mimic important features of the complexity of neoplastic growth [44]. Hence, we used a 3D cell culture model where multicellular aggregates (spheroids) were made using the liquid overlay technique [45]. After 4 days in 3D cell culture conditions, WT spheroids showed a well-rounded spheroid phenotype (Fig. 5A). Strikingly, in 3D cell culture, DMT1 ablation significantly increases the presence of dispersed cells at the periphery of the spheroid, a phenotype that was significantly rescued by DMT1 re-expression in the DMT1 KO cells (Fig. 5A). No significant differences in cell viability were observed upon DMT1 silencing in either MDA-MB-231 or T47D breast cancer cells (Figure S2F). Optical Coherence Tomography (OCT) imaging showed that DMT1 silencing led to the generation of multicellular aggregates with lower height and sphericity and a more ellipsoidal shape with a clear dispersion of cells at the periphery in comparison to WT spheroids (Fig. 5B). Furthermore, using a ratiometric oxygen nanoparticle biosensor [46], we established that MDA-MB-231 WT spheroids display the presence of a hypoxic core, which is a common feature of tumors. Interestingly, we observed that DMT1 silencing disrupted the formation of a hypoxic core in the multicellular aggregates. Instead, DMT1 KO spheroids displayed a less regular O_2_ distribution with lower overall oxygenation (Fig. 5C). These results are consistent with the role of DMT1 in mitochondrial oxygen consumption (Fig. 4).

Next, we analyzed global transcriptomic changes in WT versus DMT1 KO MDA-MB-231 breast cancer cells in both 2D and 3D cell culture conditions. For 2D culture, we found a total of 253 differentially expressed genes upon DMT1 silencing in MDA-MB-231 cells (65 upregulated and 188 downregulated), and for 3D multicellular aggregates, we observed a total of 296 differentially expressed genes upon DMT1 silencing in MDA-MB-231 cells (143 upregulated and 153 downregulated), when we used the cutoff ±2 log2FoldChange and adjusted *p*-value < 0.05 in the DEG analysis. These results were represented in volcano plots as shown in Fig. 5D. Circos plot shows the overlap of both genes and GO terms from 2D (magenta) and 3D (green) DMT1 KO-regulated gene lists (Fig. 5D). The group of genes shared between both lists is colored in dark orange while unique ones are colored in light orange. Purple-linked lines connecting dark orange arcs depict the overlap extent between the two gene lists. Interestingly, the blue lines, which link the different genes falling into the same ontology term, are highly represented in the Circos plot suggesting functional overlap between genes regulated by DMT1 in either 2D or 3D cell culture conditions (Fig. 5D). Then, to evaluate the regulation of mutual pathways between 2D DMT1 KO and 3D DMT1 KO differentially regulated gene lists, we generated a GO clustered heatmap (Fig. 5E). Extracellular matrix organization, metal ion response, negative regulation of endopeptidase activity, positive regulation of cell migration, and chemotaxis are among the significantly enriched pathways in 3D compared to 2D cell culture conditions upon DMT1 silencing. Interestingly, an integrated system for motif activity response analysis (ISMARA), that evaluates computationally predicted regulatory sites for transcription factors, revealed that overall transcription factor activity is conserved among 2D and 3D cell cultures upon DMT1 silencing (Fig. 5F). NRF1 transcription factor activity was the top increase upon DMT1 silencing in MDA-MB-231 cells in both 2D and 3D cell culture conditions (Fig. 5F). NRF1 can confer therapeutic resistance by triggering the proteasome bounce-back response [47]. Other common upregulated transcription factor activity includes YBX1, associated with aggressiveness in breast cancer [48, 49], and ZBTB33 (also known as Kaiso) which had been associated with metastatic activity in MDA-MB-231 cells [50]. In contrast, among the top shared-decreased transcription factor activity upon DMT1 KO was REST whose loss is associated with aggressive breast cancer [51], and HIF2A that contrary to HIF1A does not affect pulmonary metastasis [52], and interestingly is more associated with long-term hypoxic compared to HIF1A [53].

To further analyze the DMT1-dependent transcriptome regulation in 3D we generated Venn diagrams of either upregulated or downregulated genes only in 3D and not in 2D. We obtained a total of 60 upregulated and 72 downregulated genes in 3D cell culture upon DMT1 ablation (Fig. 5G). GO analysis of this subset of genes confirmed that terms, such as matrisome components and chemotaxis, were enriched in genes regulated exclusively in 3D upon DMT1 silencing (Fig. 5G). Lastly, we clustered gene expression data for each sub-group evaluated i.e., 2D WT, 2D DMT1 KO, 3D WT, and 3D DMT1 KO by the normalized gene expression levels of both hypoxia and iron transport gene signature, and EMT and dormancy gene signature as important processes regulating metastatic behavior (Fig. 5H). A clustered heatmap of significantly altered gene expression revealed that 18 genes were significantly regulated in both hypoxia and iron transport gene signatures [54, 55]. Interestingly the hypoxia signature appears to be upregulated in 3D upon DMT1 silencing (elevated number of upregulated genes), which is in accordance with our results using the oxygen probe MMIR1 (Fig. 5C), and conversely, the iron transport signature seems downregulated in 3D compared to 2D cell culture condition upon DMT1 ablation. For example, VEGFA from hypoxia and LCN2 from iron transport gene signatures were upregulated in 2D and downregulated in 3D upon DMT1 silencing, indicating that important differences in particular genes can occur in opposite ways depending on whether they are evaluated in the context of 2D or 3D cell culture. Moreover, the clustered heatmap analyses of EMT gene signature showed that most of this set of genes was downregulated upon DMT1 silencing. On the other hand, the dormancy gene signature was half downregulated and half upregulated upon DMT1 silencing in MDA-MB-231 cells growing in 3D cell culture. Overall, these results reveal that DMT1 may promote invasiveness and aggressiveness in 3D culture conditions by regulating hypoxia, iron transport, EMT, and dormancy cancer-related processes at the transcriptional level.

### Reduced DMT1 levels are associated with metastatic progression in breast cancer

Since DMT1 silencing appears to result in a novel iron phenotype that is also found in metastasis-derived triple-negative breast cancer cell lines, we have evaluated the role of DMT1 in breast cancer metastasis. Using TNMplot, an integrated database of available transcriptome-level datasets enabling the comparison of normal, tumor, and metastatic data across all genes [56], we observed that DMT1 gene expression was significantly decreased in metastatic tissue compared to primary tumor in breast cancer patient samples as well as in esophageal and prostate cancer. However, in some types of cancers such as colon and kidney cancer DMT1 was upregulated or not regulated in metastatic tissue, respectively (Fig. S4A). Moreover, we have evaluated the role of DMT1 in breast cancer cell metastasis using single-cell transcriptomic datasets from patient-derived xenografts (PDX) models of triple-negative breast cancer [57]. Interestingly, we observed that DMT1 expression was significantly reduced in lung distant metastases compared with primary tumors (Fig. S4C). Finally, analyses of estrogen receptor positive (ER+) and ER- breast cancer patients showed that DMT1 gene expression downregulation correlates with lower survival in ER-, but in ER+ patients lower DMT1 expression correlates with higher survival (Fig. S4B). However, contradictory results were found when we investigated the clinical association between DMT1 and breast cancer, using data from annotated patient datasets of a normal breast tissue cohort in comparison with cohorts of breast tumor tissue. We found a significant increase in DMT1 mRNA expression in tumors compared to normal breast tissue samples (Fig. S4D). Furthermore, Kaplan Meier survival analysis of the invasive breast carcinoma patient TCGA dataset showed that higher expression of DMT1 is significantly associated with lower overall survival (Fig. S4E). These results indicate that although high expression of DMT1 is detected in primary tumors, DMT1 expression is downregulated once these cells reach the metastatic site and undergo metastatic growth being specifically associated with triple-negative breast cancer phenotype. An alternative hypothesis is that DMT1 may not be necessary for metastasis, and therefore it is negatively selected for in metastatic cells.

### DMT1 silencing in MDA-MB-231 cells increases lung metastasis overgrowth in vivo

**Fig. 6:**
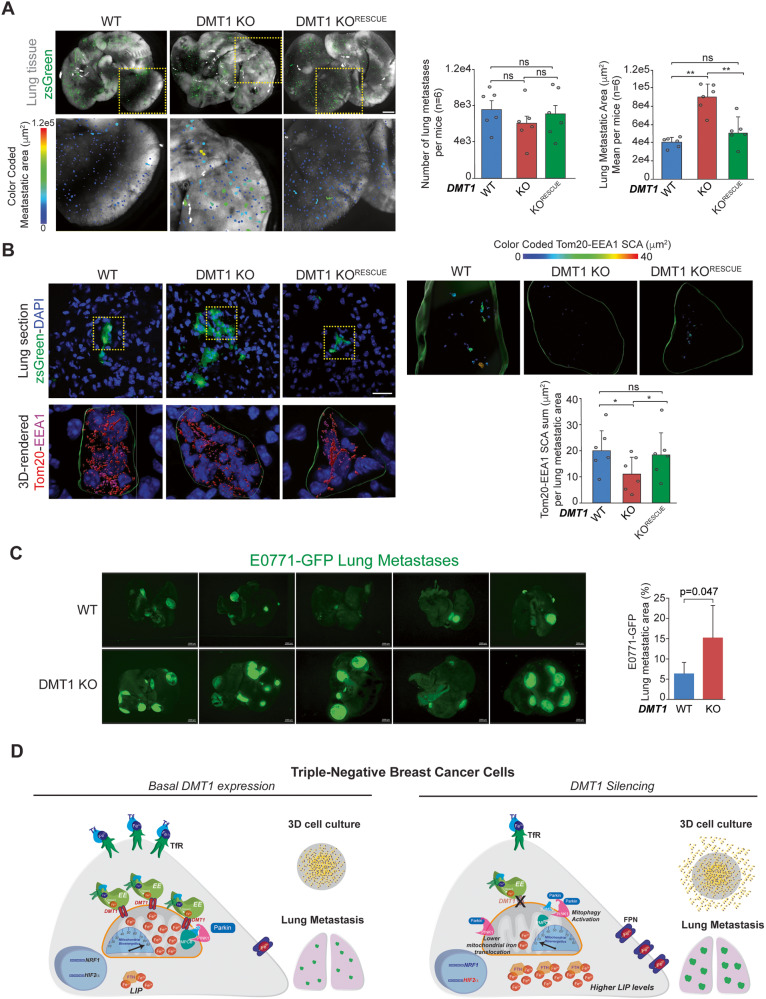
DMT1 silencing increases the lung metastatic outgrowth of triple-negative breast cancer cells in vivo. **A Left**, images of lungs harvested after three weeks from NSG mice tail vein injected with ZsGreen labeled MDA-MB-231 WT, DMT1 KO, and DMT1 KO^RESCUE^ cells. **Right**, bar graphs show the number and the area of lung metastatic lesions in each condition (*n* = 6 mice per condition). Scale bar = 2 mm. **B Left**, immunofluorescence of Tom20 (red) and EEA1 (magenta) in lung micrometastasis cryosections of mice evaluated in **A**. **Right**, the sum of SCA between both markers per lung metastasis in 6 lesions per condition was color-coded (top) and quantified (bottom). **C** **Left**, images of lungs harvested after three weeks from C57BL/6 mice tail vein injected with GFP-labeled E0771-GFP cells. **Right**, bar graph shows the area of lung metastatic lesions in each condition (*n* = 5 mice per condition). An unpaired *t*-test was used for comparison between the two conditions. Scale bar = 2000 μm. **D** Working model. DMT1 regulates LIP levels and metastatic outgrowth in triple-negative breast cancer cells. DMT1 downregulation alters iron metabolism by preventing adequate iron translocation into the mitochondria, inducing metabolism alterations, and mitophagy activation through its molecular partner PMPCB-related mechanism, ultimately leading to enhanced metastatic outgrowth in the lung in vivo. This mechanism provides a rationale for a targeted iron metabolism disruption/restoration that may be translated therapeutically.

To test whether DMT1 is detrimental for metastasis progression, we evaluated metastatic growth using an in vivo lung metastatic model. ZsGreen-labeled MDA-MB-231 cells were intravenously injected in NSG mice, and after 3 weeks, lungs were harvested and processed for in situ imaging of lung tissue. Strikingly, we observed no effect on the colonization capacity of MDA-MB-231 cancer cells upon DMT1 silencing, reflected by a similar number of metastases detected in the lungs of mice intravenously injected with WT or DMT1 KO cells (Fig. 6A). However, the size of the metastases was significantly increased upon DMT1 ablation, an effect that was rescued by the re-expression of mouse-DMT1 in the DMT1 KO cells (Fig. 6A). Using immunofluorescence of tissue cryostat sections, we evaluated EE-mitochondria interactions in metastatic cells in the lungs. Similarly, to the in vitro results shown in Fig. 1D, we observed that DMT1 silencing decreases the EE-mitochondria association of MDA-MB-231 cells in growing metastatic foci (Fig. 6B). These results indicate that in lung metastases, DMT1 maintains levels of EE-mitochondria interactions that are associated with decreased lung metastases growth. Importantly, when we tested E0771 cells in an in vivo mouse model of lung metastasis we observed that DMT1 ablation significantly increased the metastatic burden/area in the mouse lungs (Fig. 6C). These results indicate that DMT1 silencing in triple-negative breast cancer cells induces a novel iron phenotype, that is characterized by decreased EE-mitochondria interactions, increased LIP levels, and promotes lung metastatic outgrowth (Fig. 6D). Altogether, our results indicate that iron metabolism and its balance between the cytoplasmic organelles and mitochondria may be at the basis of a specific adaptive phenotype for the metastatic capacity of triple-negative breast cancer cells.

## Discussion

In the present work, we showed that DMT1 silencing disrupts iron homeostasis in triple-negative breast cancer cells by decreasing EE-mitochondria interactions, reducing mitochondrial iron translocation, increasing LIP levels, and establishing a compensatory iron phenotype of elevated export/reduced import. Concomitantly, DMT1 ablation results in the induction of PINK1/Parkin-dependent mitophagy, via the peptidase PMPCB, a recently described DMT1 interactor, that regulates PINK1 turnover at the mitochondria. These results suggest that DMT1 either directly or indirectly via its role in mitochondrial iron translocation plays an important role in the intracellular organization and function of EE and mitochondria organellar compartments.

One important metabolic characteristic acquired by several cancer types it’s the so-called iron-seeking phenotype [8]. Cancer cells are expected to require higher levels of iron to support their growth and proliferation requirements [8]. In MDA-MB-231 cells, DMT1 silencing downregulates TfR, a well-known marker for ferroptosis [58], without affecting cell viability, suggesting that ferroptosis is not among the cellular processes regulated by DMT1 in breast cancer cells. Moreover, DMT1 ablation upregulates FPN and iron storage protein FTH, likely reflecting a compensatory response to the LIP increase also induced by DMT1 ablation. Recently, DMT1 silencing in head and neck cancer cells has been shown to result in increased levels of TfR protein/mRNA as well as elevated levels of LIP [59]. One possibility to reconcile these results is that as shown for T47D ER+ breast cancer cells, head and neck cancer cells display a different DMT1-mediated regulation of iron metabolism. This heterogeneity in DMT1 function across different cancer types is an important observation that needs to be accounted for when studying iron metabolism in cancer and metastasis progression.

In summary, we show that in DMT1-depleted MDA-MB-231 cells, LIP levels and mitochondrial ROS levels are increased while chelatable mitochondrial iron levels are decreased. Previously, it has been shown that higher levels of redox-active mitochondrial LIP can act as a catalyst in the generation of ROS in this organelle [60]. In contrast, we propose that reduction of mitochondrial iron translocation can lead to mitochondrial dysfunction that has been described to result in increased mitochondrial ROS production [33]. Moreover, another group demonstrated that mitochondrial targeted deferoxamine (iron chelator) induces a reduction in mitochondrial LIP leading to increases in the mitochondrial superoxide production [32], analogous to what we observe upon DMT1 silencing.

DMT1 silencing appears to shift the oncogenic high-TfR/low-FPN phenotype of MDA-MB-231 cells to a low-TfR/high-FPN breast tumor suppressor phenotype [61]. These results are consistent with the elevated DMT1 mRNA expression in tumors compared to normal breast tissue samples also associated with lower overall survival in breast cancer patients. However, the combination of the increasing LIP and ROS levels via mitochondrial iron translocation blockage and diminishing mitochondrial oxygen consumption and glycolysis upon DMT1 silencing could be at the basis of the acquisition of enhanced outgrowth metastatic capacity upon DMT1 ablation in MDA-MB-231 breast cancer cells. Strikingly, these effects in LIP levels and lung metastatic overgrowth are achieved by a highly specific and targeted disruption of DMT1-dependent iron metabolism in both human and murine triple-negative breast cancer models, i.e., MDA-MB-231 and E0771, respectively.

The conflicting set of results across normal, tumor, and metastatic tissue gene expression as shown in Fig. S4, together with our experimental results suggests a complex role for DMT1 in tumor and metastatic growth. Moreover, DMT1 silencing affects cancer cell processes, such as cell aggregation/compaction, mitochondrial metabolism, and oxygenation in multicellular aggregates. Transcriptomic profiles upon DMT1 silencing are strikingly different between 2D and 3D culture conditions, suggesting that the cellular environment influences the phenotype developed by DMT1 KO MDA-MB-231 cells. Such differences indicate an important fitness adaptation to the 3D environment, with DMT1 silencing being an advantage for metastatic outgrowth in physiological contexts in triple-negative breast cancer cells. Notwithstanding, it is well-recognized that 3D cell culture conditions can more accurately recapitulate tumoral characteristics [44]. The results from the in vivo lung metastasis assay showing that DMT1 silencing promotes the growth of metastatic nodules upon lung colonization and the data obtained from PDXs triple-negative breast cancer models as well as a comprehensive database comparing normal, tumor, and metastatic tissue, strongly support 3D cell culture as a representative readout to evaluate cancer-related processes. Interestingly, in both in vitro and in vivo lung metastases, EE-mitochondria associations decrease in MDA-MB-231 cells upon DMT1 ablation. In summary, we propose a role for DMT1 in metastatic outgrowth, but not colonization/seeding, via reduction of endosome-mitochondria interactions and subsequent disruption of iron metabolism as shown by increased LIP levels.

A recent study reported that DMT1 was downregulated in the metastases from PDX models similar to our analysis performed with the same single-cell RNAseq dataset in Fig. S4B [62]. In this work, TfR is downregulated in metastases mimicking the effect that we observed upon DMT1 silencing in MDA-MB-231 cells, suggesting that iron-transporter genes are suppressed in metastatic cells compared to primary tumors. Moreover, our data from lung and brain metastatic-derived MDA-MB-231 cells show a significant decrease in mitochondrial iron translocation and an increase in LIP levels in these cells, compared to parental MDA-MB-231 cells. An interesting hypothesis is that a simultaneous reduction in mitochondrial iron transport dependent on EE-mitochondria interactions and a buildup in cytoplasmic LIP may lead to increased oxidative stress and subsequent metabolic adaptations favorable to metastatic outgrowth.

MDA-MB-231 and T47D breast cancer cells are representatives of two different types of breast cancer. MDA-MB-231 is a well-characterized cellular model of triple-negative breast cancer, showing a mesenchymal phenotype, which is consistent with their capacity to invade in vitro and to generate spontaneous metastases when orthotopically implanted in immunodeficient mice [63]. Conversely, the luminal A breast cancer cell line T47D, forms highly cell-cell adhesion cohesive clusters, representative of a more epithelial phenotype. Importantly, T47D’s ability to form large and aggressive tumor xenografts depends on the presence of estrogen [63], and in cell culture, in the absence of estrogen, T47D is commonly denoted as a non-invasive cancer cell line [64–66]. Previously, we have shown that T47D cells grow adequately both in 2D cell culture, 3D spheroids, and tumor xenografts independently of estrogen [28, 67–70]. Moreover, we have shown significant differences in receptor-mediated endocytic trafficking pathways, iron metabolism-related processes and transferrin uptake into tumor xenografts between T47D and MDA-MB-231 in the absence of estrogen [28, 67–70]. Therefore, since our main goal is to compare iron-related and cancer cell-related processes in MDA-MB-231 and T47D breast cancer cell lines, we have consistently grown these two cell lines in the absence of estrogen. Interestingly, although MDA‐MB‐231 cells show lower DMT1 expression in comparison to T47D cells, DMT1 silencing in MDA-MB-231 has a greater effect in endosome-mitochondria interactions, iron and mitochondria metabolism. One possibility is that this difference between MDA-MB-231 and T47D cells is due to activating mutations, ERK activation/mutant p53 and PI3K activation, respectively. We speculate that lower DMT1 expression in ER+ cells (such as T47D) or breast cancer patients can correlate with higher survival. However, in triple-negative breast cancer cells lower DMT1 expression appears to correlate with an aggressive metastatic phenotype. These complex regulations between different types of cancer even from the same primary type highlight the urgent need for systematic analyses of oncogenic/tumor suppressor factors using different breast cancer types and tumor models.

Our previous work demonstrated a remarkable difference between these two cell lines in terms of iron-related protein expression, endosomal trafficking, transferrin recycling, and endosomal iron retention/release properties [28, 70]. Here, we showed a differential DMT1 requirement for both EE-mitochondria interactions and mitochondrial iron translocation in MDA-MB-231 and T47D breast cancer cells. In comparison with our previous work [12], we have expanded our live cell imaging approach by both visualizing and quantifying EE-mitochondria interactions in 3D using surface contact area (SCA) analysis in z-stacks images. We found that DMT1 regulates EE-mitochondria “kiss-and-run” transient interactions as well as EE dynamics, and mitochondrial iron translocation levels in MDA-MB-231 but not in T47D cells. Previously, we have shown that, in epithelial cells, a mutant form of Tf, which cannot release iron (Lock-Tf), increased endosomal speed, independently of EE-mitochondria interaction [12]. Moreover, MDA-MB-231 cells show a delay in the iron release from Tf in endosomes as compared to T47D [70]. Herein, we show that DMT1 ablation blunted endosomal speed in MDA-MB-231 breast cancer cells. Since Tf is “upstream” of DMT1 in the process of iron transport into the mitochondria, after being released from Tf, iron needs to be reduced to its ferrous form to become available for DMT1-mediated transport. Our experiments using a LIP biosensor indicate that ferrous iron accumulates in endosomal-like structures of MDA-MB-231 cells upon DMT1 silencing, as shown previously in HepG2 cells [71]. Together, our results suggest a correlation between endosomal speed and the iron status of the endosomal lumen. We speculate that ferric iron accumulation in the endosomal lumen [12, 28, 70], leads to increased endosomal speed, whereas high ferrous iron levels correlate with reduced endosomal speed. The mechanism involved in these processes is still unknown but could be related to the way the endosomal lumen redox environment connects with the molecular motors and/or the adapter proteins governing endosomal motility.

Previously, it has been suggested that epithelial mammary cells and triple-negative breast cancer cells display divergent mechanisms controlling iron-related processes [72, 73]. Our findings indicate that MDA-MB-231, but not T47D cells, depend on DMT1 for mitochondrial iron translocation, indicating the existence of distinct iron transport pathways among different breast cancer cell lines. Whereas in MDA-MB-231 cells DMT1 regulates mitochondrial iron transport via endosome-mitochondria interactions, in T47D cells other intracellular iron transporters such as PCPB1–2 and Zip14 [30, 74], and putatively lysosome-mitochondria interactions [75] may mediate mitochondrial iron translocation. These results suggest a differential requirement for DMT1 in the maintenance and regulation of iron metabolism across different breast cancer cell lines. Our previous work showed that in MDA-MB-231, endosomes are larger and more peri-nuclear than in T47D cells [28]. Interestingly, mitochondria also tend to be concentrated in the perinuclear region in MDA-MB-231 cells. The notable differences in endosomal characteristics between these two breast cancer cell types can be explained by numerous variables such as genetic background, level of differentiation, the differential activity of enzymes and metabolic pathways, or presence of mutations in signaling pathways such as PI3K, that are important for endocytic trafficking and regulation of endosomal maturation [76]. Nevertheless, an interesting hypothesis is that the different intracellular distribution of endosomes and mitochondria within the two breast cancer cell lines analyzed in this study can be the basis of a novel invasive cancer cell’s vulnerability related to iron homeostasis.

PINK1/Parkin-mediated mitophagy can regulate breast cancer malignancy [39]. Interestingly, iron chelation specifically increases the expression of FTMT and the translocation of its precursor to the OMM, inducing mitophagy and suppressing tumor development [37]. Moreover, it has been recently described that mitochondrial iron chelation activates mitophagy, impairing mitochondrial metabolism and tumoral growth both in vitro and in vivo [32]. Mitochondrial-targeted deferoxamine (mitoDFO), induces PINK1-dependent mitophagy in hormone-dependent MCF7 and triple-negative MDA-MB-231 breast cancer cells [32]. Notably, our results show that DMT1 silencing induces PINK1/Parkin-dependent mitophagy as well as FTMT in MDA-MB-231 but not in T47D breast cancer cells. Our results indicate that contrary to these previous reports, mitophagy hyperactivation can enhance malignancy, specifically metastatic capacity. Sandoval-Acuña et al. [32], showed only slight differences in TfR and FPN expression upon mitoDFO treatment, suggesting that LIP in the cytosol is not significantly affected by mitoDFO. In contrast, DMT1 ablation by specifically disrupting EE-mitochondria interactions can simultaneously decrease mitochondrial iron translocation and increase LIP levels and enhance metastatic outgrowth.

Here, we have demonstrated a critical requirement of DMT1 for an adequate mitochondrial metabolism as well as metastatic capacity. We propose that DMT1’s role in EE-mitochondria transient interactions, mitochondrial iron translocation, mitophagy, and cellular iron metabolism/transport regulation, is key to the adaptation of invasive breast cancer cells to the metastatic niche, enhancing their metastatic outgrowth. DMT1 silencing lowers mitochondrial metabolism, suggesting a rewiring and/or adaptation to reduced iron levels in mitochondria in combination with higher levels of LIP in the cytoplasm. Considering the in vivo and 3D cell culture experiments, this iron-mediated rewiring of mitochondrial metabolism may be associated with increased aggressiveness and metastatic behavior of triple-negative breast cancer cells. We posit that a complex DMT1-mediated relationship between iron-related processes and mitochondrial metabolism may be at the basis of metastatic disease progression in breast cancer. Future experiments could corroborate the hypothesis of a specific role of DMT1 as a particular and highly specific Achilles’ heel for triple-negative breast cancer cells during metastasis.

## Materials and methods

### Cell culture

MDA-MB-231, T47D, and HEK293T cell lines were obtained from ATCC, and MDA-MB-231-TGL parental, MDA-MB-231-TGL LM2 and MDA-MB-231-TGL BrM2 were obtained from Dr. Joan Massague laboratory (Memorial Sloan Kettering Cancer Center, NY, USA). All cell lines were grown at 37 °C in a humidified atmosphere containing 5%-CO_2_. Cells were cultured in DMEM (Thermo Fisher Scientific) or RPMI 1640 (for murine triple-negative breast cancer cell line E0771-GFP) supplemented with 10% fetal bovine serum (FBS) (ATCC), 100 units/ml penicillin, 100 ug/ml streptomycin, and 4 mM L-glutamine. Cells were used at passage numbers equal to or lower than 10 and tested routinely for mycoplasma contamination using PCR.

### CRISPR/Cas9 mediated DMT1 silencing

The silencing of DMT1 in MDA-MB-231, T47D, and E0771 cell lines was performed using lentiCRISPRv2 plasmid containing the DMT1 (SLC11A2) targeting sgRNA sequence 5’-TGAGAAGATCTCCATTCCTG-3’. Lentiviral particles were produced in HEK293T cells by transfection with 0.2 µg of pCMV-VSV-G (Addgene), 2 µg of psPAX2 (Addgene) and 2 µg of lentiCRISPRv2 sgRNA DMT1. After 48 h, the supernatant of transfected cells was collected and filtered using a 0.45-µm PES filter. The lentiviral suspension was immediately used for transduction or stored at −80 °C. A 1:1 lentiviral vector suspension containing 10 µg of polybrene (SCBT) was added drop by drop to either MDA-MB-231 or T47D cells and incubated for 72 h. For antibiotic selection, 1 µg/mL of puromycin (Gibco) was used. The cells were cultured until all the non-transduced control cells died (2–3 days) plus 2 more days in antibiotic selection media. For rescue of DMT1 expression in MDA-MB-231 DMT1 KO, cells were transfected with plasmid pEGFP-C1-DMT1-GFP (N-terminal GFP, DMT1 isoform 2, kindly provided by Dr. Jerry Kaplan, Department of Pathology, University of Utah Health Sciences Center, Salt Lake City, UT) that had been previously used in human cancer cells [77]; then cells were further selected with Neomycin (100 μg/ml) until all non-treated control cells died (2 weeks) plus 2 more days in antibiotic selection media.

### Indirect immunofluorescence

Immunofluorescence experiments were performed as previously described [28]. Briefly, cells were seeded into Ibidi 8-well μ-plates (Ibidi, #80827) and grown overnight at 37 °C in a 5% CO_2_ environment. Cells were fixed for 15 min with 4% paraformaldehyde (PFA) at 37 °C, permeabilized with 0.2% TritonX-100 in PBS for 15 min at room temperature, blocked for 90 min on a gentle rocker-shaker in blocking buffer (1% bovine serum albumin (BSA), 0.5% fish skin gelatin (FSG), 0.1% Triton X-100), and immunostained with primary antibodies overnight at 4 °C, then incubated with secondary antibodies for 1 h at room temperature, post-fixated with 4% PFA for 10 min, and stained with DAPI (1μg/ml) for 15 min. All solutions were 0.45 μm syringe filtered. Images were acquired using Thunder microscope (Leica) with oil immersion 63X/NA 1.4 objective, subsequently subjected to integrated computational clearing deconvolution process (LASX software), and then 3D-rendered and further analyzed using Imaris 9.6 software (Bitplane). Colocalization Pearson coefficient was calculated using Imaris integrated “coloc” tool [28].

### Live cell imaging

For live cell imaging, cells were seeded in N° 1.5 glass-bottom 35-mm Petri dishes coated with poly-D-lysine (MatTek Corporation). Imaging medium comprised phenol red–free and serum-free DMEM supplemented with 4 mM L-glutamine. Live mitochondrial and EE staining was achieved with 100 nM MitoTracker Red (Molecular Probes) and a 2-min pulse of 50 µg/ml Tf AF488 (Thermo Fisher Scientific), respectively. Before imaging, the cells were briefly washed and chased with imaging media for another 2 min. Starting at time point 5–6 min after Tf pulse, time-lapse videos (4 frames per second; 5 z-stack series) of 45-s duration were acquired. The assay was performed under CO_2_ and temperature control within the live-cell incubation chamber of the Thunder microscope (Leica). Mitochondrial iron translocation was measured by incubating live cells with 100 nM iron sensor dye rhodamine B 4-[(2,20-bipyridin-4-yl)-aminocarbonyl] benzyl ester (RDA) [12, 29] (a gift from Dr. Ursula Rauen, Institute of Physiological Chemistry, University Hospital Essen, Essen, Germany) for 15 min at 37 °C in a cell incubator, and then subjected to live cell imaging for 5 min at a time interval of 5-s performed under CO_2_ and temperature-controlled incubation chamber of the Thunder microscope using oil immersion 63X/NA 1.4 objective (Leica). Labile iron pool, mitochondrial superoxide levels, and mitochondrial membrane potential were evaluated by fluorescence imaging using FerroOrange (Dojindo), MitoSOX Red (Thermo Fisher Scientific), and tetramethylrhodamine ester TMRM (Thermo Fisher Scientific), respectively. Briefly, live cells were plated as described above and incubated at 37 °C with either 1 μM FerroOrange, 1 μM MitoSOX, or 100 nM TMRM 100 for 30 min, respectively. Live cells were imaged using the Thunder microscope with oil immersion 63X/NA 1.4 objective (Leica). Fluorescence dequenching of RDA and mean intensity of FerroOrange, MitoSOX red, or TMRM was analyzed in 40 cells per condition using ImageJ software (NIH).

### 3D rendering and surface contact area (SCA)

Images from indirect immunofluorescence and live-cell imaging z-stack series experiments were 3D rendered using Imaris 9.6 (Oxford Instruments). Briefly, the surface of mitochondria was determined according to either the Mitotracker or anti-Tom20 immunostaining, while EE structures were obtained from 2 min-Tf uptake data. DMT1 surface was obtained from anti-DMT1 immunostaining. For SCA measurements between organelle markers, the XTension “Surface Surface Contact Area” IMARIS plugin (https://imaris.oxinst.com/open/view/surface-surface-contact-area) was used. The morphology properties of EE, mitochondria, DMT1, and SCA properties were measured using Imaris 9.6 surface module. Live-cell imaging data from 10 rendered cells using 5 consecutive time lapses for each experimental condition (50 technical replicates) was analyzed.

### Immunoblotting

Cells were lysed in immunoblotting buffer (25 mM HEPES, 150 mM NaCl, 1 mM MgCl_2_, 0.4% NP-40, pH 8), supplemented with anti-protease and anti-phosphatase mixture (Millipore) at 4 °C. Protein lysates were clarified by centrifugation. Protein lysates for western blotting were separated by SDS-PAGE, transferred to PVDF membranes (Bio-Rad), and then subjected to standard immunoblotting procedures as previously described [28, 78].

### Cellular bioenergetics

Cells were seeded (2 × 10^4^ cells/well) in XF96-well microplates (Agilent Technologies) and cultured at 37 °C 5% CO_2_ for 24 h. The day before the assay, XF sensor cartridges were hydrated. One hour before the assay, culture media was replaced with DMEM XF base medium pH 7.4 supplemented with glutamine and glucose (Sigma-Aldrich). Oxygen consumption rate (OCR) and extracellular acidification rate (ECAR) were measured at 37 °C on an XF96 Extracellular Flux Analyzer (Agilent Technologies) using the Cell MitoStress Test Kit (Seahorse Bioscience), according to manufacturer instructions. The baseline (basal) OCR was measured three times before, and then three times after each sequential injection of oligomycin (1 μM), carbonyl cyanide 4-(trifluoromethoxy) phenylhydrazone (FCCP) (0.5 μM), and rotenone and antimycin A (both 0.5 μM) and this last injection port was used also for nuclei stain using Hoechst. Upon completion of the assay, the microplate was imaged using Cytation5 microplate reader (BioTek) for nuclei stain detection. The assay was normalized by the number of cells in each well. Data generated by the XF Extracellular Flux Analyzer (Agilent Technologies) were exported and analyzed using Microsoft Excel.

### Liquid overlay spheroid fabrication and imaging

Cell monolayers were detached from their culture flask via standard trypsinization protocol. Cells were counted and re-suspended in media to the desired concentration of 2.5 × 10^5^ cells/mL. Next, the cell suspension was dispensed into the round bottom, non-adherent, 96 well-plates (CellStar, Greiner Bio-One) at a volume of 100 μL per well. These suspensions were cultured with a concentration of 2.5% Matrigel. Plates were centrifuged for 10 min at 1 000 rpm, immediately following seeding to ensure collection of the cell pellet at the bottom of the well. Plates were then incubated, and cell aggregates were cultured over a four-day maturation period. Spheroids were imaged using the Leica Thunder microscope (N PLAN 5 x / 0.12 PH 0 Dry objective) by phase contrast imaging on day 4 and then fixed with PFA 4% for Optical Coherence Tomography (OCT) imaging. The relative cellularity outside the spheroid core was calculated as the area with cells outside the core of each spheroid using ImageJ (NIH).

Spheroids oxygenation was analyzed using the O_2_-sensitive MMIR1 nanoparticle probe, as described previously [46, 79], with minor modifications. Briefly, cells were incubated with the O_2_ probe at 5 μg/ml for 4 days in a cell incubator (at 37 °C in a humidified atmosphere containing 5%-CO_2_), and then imaged using the Leica DMi8 Thunder microscope (N PLAN 5 x / 0.12 PH 0 Dry objective) using for red reference (Filter cube DFT51010 exc. = 555 nm, em. range= 590 nm) and far-red O_2_-sensitive (Filter cube DFT51010 exc. =635 nm, em. = 700 nm) spectra. The intensity data were obtained using ImageJ (NIH) after 50 pixels background subtraction and the O_2_ probe intensity ratio (R) was calculated using the formula R = I_ref_/I_sens_.

For OCT imaging, a commercial Spectral Domain Optical Coherence Tomography (SDOCT) system, operating with a central wavelength of 1310 nm (TEL220C1, Thorlabs Inc.) was utilized [45, 80]. The SDOCT system has an axial resolution of 5.5 and 4.2 μm in air and water, respectively, and a lateral resolution of 5 μm. Image collection for this study was performed using previously established methods for tumor spheroid imaging [45], at a 5.5 kHz A-scan rate with a sensitivity of 101 dB. Indices of refraction used in this study were 1.33 for samples in liquid medium. Lateral pixel size was held constant at 1.0 μm.

### Experimental lung metastasis assays

MDA-MB-231 cells were labeled by stable transduction with a lentivirus expressing pHAGE-IRES-ZsGreen [81]. A total of 5 × 10^4^ cells were injected into the tail-vein of female, five-weeks-old NSG™ mice (Jackson Laboratories). Lungs were harvested 21 days post-injection and imaged using Leica M205 FA & LeicaDCF3000 G (Leica) using GFP and bright field filters. Imaris 9.6 software was used to quantify the number and area of lung metastases. All animal work was approved by the Institutional Animal Care and Use Committee at Albany Medical College (project number: 20-05002). Cryostat sections were obtained from fixed mice lungs used in this experiment and then processed for immunofluorescence and analyzed using Imaris 9.6 software (Oxford Instruments) as described above.

A total of 2 × 10^5^ E0771-GFP cells (WT and DMT1 KO) were resuspended in cold DPBS calcium, and magnesium (Thermo Fisher Scientific) and intravenously injected via the lateral tail vein in C57BL/6 mice (*n* = 5 mice per condition). This experiment was conducted in accordance with the National Institutes of Health guidelines and was approved by the Icahn School of Medicine at Mount Sinai IACUC committee. Three weeks post-injection mice developed lung metastases. The presence and area of lung metastases were evaluated using widefield fluorescence microscopy as described above and then images were quantified using ImageJ (NIH) software.

### RNA sequencing and genomic data analysis

Total RNA was isolated in triplicate from MDA-MB-231 WT and MDA-MB-231 DMT1 KO using TRIzol according to manufacturer instructions. Quality control of RNA determines that the RIN number of all 6 samples processed was >8.6. RNA library preparation was performed using a polyA selection method. RNA sequencing was performed using the Illumina HiSeq system in a 2 ×150-bp configuration (single index, per lane) by GENEWIZ. FastQC quality check analysis of the MDA-MB-231 WT and DMT1 KO samples in triplicate achieved a high-quality score. Upon receipt of the FASTQ files, the files were trimmed using Trim Galore (GitHub repository, https://github.com/FelixKrueger/TrimGalore). Reads were aligned to the hg38 genome using Rsubread v1.5.3 [82]. Gene counts were quantified by Entrez Gene IDs using featureCounts and Rsubread’s built-in annotation. Gene symbols were provided by NCBI gene annotation. Genes with count-per-million above 0.5 in at least 3 samples were kept in the analysis. Differential expression analysis was performed using limma-voom [83]. Significance was defined as *P* value < 0.05 after adjustment for multiple testing. Principal component analysis (PCA) indicates a distinct gene expression profile between conditions analyzed. Other components of this analysis account for less than 1% of the variance. Functional enrichment analysis was performed using WebGestalt (WEB-based Gene Set Analysis Toolkit, http://www.webgestalt.org) [84], and Metascape (version 3.5) [85]. ISMARA (Integrated System for Motif Activity Response Analysis) [86], was employed to estimate the key transcription factors associated with the observed differential gene expression of our RNA sequencing data from MDA-MB-231 WT and MDA-MB-231 DMT1 KO cells in both 2D and 3D cell culture conditions.

### Statistical analyses

One-way ANOVA with Bonferroni post-hoc test or unpaired two-tailed Student *t*-test was used for experimental data analysis. Data are presented as mean with a 95% confidence interval or SD was indicated. All experiments were performed with a minimum of three independent replicates. The number of cells analyzed for imaging analyses is indicated in respective figure legends. *p*-values less than 0.05 were considered statistically significant (*p < 0.05; **p < 0.01). Data were analyzed using GraphPad Prism 6.0 or Microsoft Excel, and data visualization was generated using the PlotsOfData web app [87] and Adobe Illustrator (Adobe).

### Supplementary information

Figure S1

Figure S2

Figure S3

Figure S4

Supplementary information

## Data Availability

The materials described in this manuscript, including all relevant raw data, will be freely available to any researcher wishing to use them for non-commercial purposes, without breaching participant confidentiality.
