# Supplementary material for: DMT1-dependent endosome-mitochondria interactions regulate mitochondrial iron translocation and metastatic outgrowth
Source: Oncogene. Author manuscript; Available in PMC 2024 Mar 8. (PMC10890933; doi:10.1038/s41388-023-02933-x)
Supplement: Supplementary material [file NIHMS1969376-supplement-Supplementary_material.pdf]

Supplementary Information

Figure Supplementary S1.

(A) Fluorescence intensity levels between EE and DMT1 in T47D and MDA-MB-231 breast cancer cells. Fluorescent data from the IF experiments shown in **Figure 1A** was analyzed. Bar graphs show the fluorescent intensity of both DMT1 in Tom20 and DMT1 in EE. Unpaired *t*-test; **p<0.05.

(B) The duration of EE-mitochondria interaction was analyzed in a subset of 15 endosomes in both parental WT and DMT1 KO MDA-MB-231 cells, as well as the Track speed means of subsets of interacting versus non-interacting EE was compared.

(C) Fluorescent images were 3D rendered and analyzed using IMARIS 9.6 software to quantify EE-Tom20 SCA sum and EE-Tom20 SCA mean (n=10 cells). Unpaired *t*-test.

(**D**) EE area sum, EE area mean, and the number of EE objects are shown in indicated cell lines.

(E) Mitochondrial area sum, mitochondrial area means, and the number of mitochondrial objects is shown in indicated cell lines. Parameters were calculated using data from 10 individual cells in 5 consecutive time intervals per condition and analyzed separately (n=50). One-way ANOVA with Bonferroni post-hoc test. **p<0.01. ns: non-significant (p>0.05).

(F) Representative images show color-coded EE Track Speed Mean in MDA-MB-231 and T47D WT and DMT1 KO cells. Endosomal track length and trajectories are depicted. MDA-MB-231 endosomal track speed mean histogram shows a higher frequency of slower EE in DMT1 KO compared to WT parental cells. T47D EE track speed mean

histogram shows a similar EE track speed mean frequency between WT and DMT1 KO cells. Scale bar = 10 μm.

Figure Supplementary S2.

(A) Representative immunoblots of DMT1 in MDA-MB-231 WT, DMT1 KO, and DMT1 KO^{RESCUE} cells. β-Actin was used as a loading control.

(**B**) EE colocalizes with DMT1-GFP. MDA-MB-231 cells were transiently transfected with pDMT1-GFP and then incubated with Tf-AlexaFluor 647 for 2 min for EE labeling. Live cells were imaged using a Leica Thunder microscope (63x oil immersion objective). Images demonstrate the co-distribution of both markers. Scale bar = 10 μm.

(C-D) Normalized fluorescence quantification of MitoSOX (C) and TMRM (D) dyes in indicated cells at steady state. Graphs show the quantification of fluorescence intensity in arbitrary units (AU). Fluorescence intensity was analyzed using ImageJ software in 40 cells per condition. One-way ANOVA with Bonferroni post-hoc test. **p<0.01. ns: non-significant (p>0.05).

(E) In T47D breast cancer cells DMT1 silencing did not alter the protein expression levels of TfR or mitophagy-related markers. (A) Immunoblots and (B) normalized densitometry quantification of TfR, PINK1, Parkin, LC3B-II, and mitochondrial ferritin (FTMT) in T47D WT and T47D DMT1 KO cells. β-Actin was used as a loading control. Unpaired *t*-test; n = 3; ns = not significant.

(F) DMT1 silencing did not affect cell viability in either MDA-MB-231 or T47D breast cancer cells. 3 000 cells were plated in 96-well plates (8-12 wells per condition) and

incubated for 3 days at 37°C in 5% CO_2. Cells were fixed with 4% PFA, permeabilized with 0.1 % Triton, and then stained with DAPI (10 μg/ml) for 10 minutes. The number of cells was counted using Cytation 5 (Biotek) according to manufacturer instructions. Unpaired *t*-test; n = 3; ns = not significant.

Figure Supplementary S3.

Additional Gene Ontology analyses from RNA seq data upon DMT1 KO in MDA-MB-231 cells 2D and 3D cell culture conditions. Functional enrichment analysis was performed using WebGestalt (WEB-based Gene Set Analysis Toolkit, http://www.webgestalt.org) [39].

Figure Supplementary S4.

(A) Violin plots of DMT1 gene expression in cancers where its expression is downregulated in metastasis i.e., breast, oesophageal, and prostate cancer (left panel), upregulated in metastasis i.e., colon and liver cancer (mid panel) and not regulated in metastatic tissue i.e., kidney and lung cancer. Data was generated using the TNMplot website tool (www.tnmplot.com). Normal, tumor, and metastatic tissue gene comparison was statistically analyzed using the Kruskal–Wallis test. The statistical significance cutoff was set at $p < 0.01$. P-values obtained from a Kruskal–Wallis post-hoc Dunn's test are indicated in each graph.

(B) Kaplan- Meier survival plots obtained from www.KMplot.com breast cancer database of both ER+ and ER- breast cancer patients (left and right panel, respectively). Hazard ratio (HR) and log-rank P values are indicated.

(C) DMT1 levels in single cells from patient-derived xenografts (PDXs) primary tumors and their matching lung micrometastases (GSE123837).

(D) 21 samples of epithelial breast tissue were compared with two breast tumor cohorts (Clynes and Shaw, 121 and 42 samples, respectively) using the R2 genomics analysis and visualization platform.

(E) High DMT1 (SLC11A2) mRNA expression is associated with decreased overall survival probability in tumor breast invasive carcinoma patients. Kaplan Meier's overall survival probability analysis of a tumor breast invasive carcinoma cohort is shown. The long-rank test result is indicated. The cut-off was calculated using the scanning method.

Supplementary Figures

Figure S1

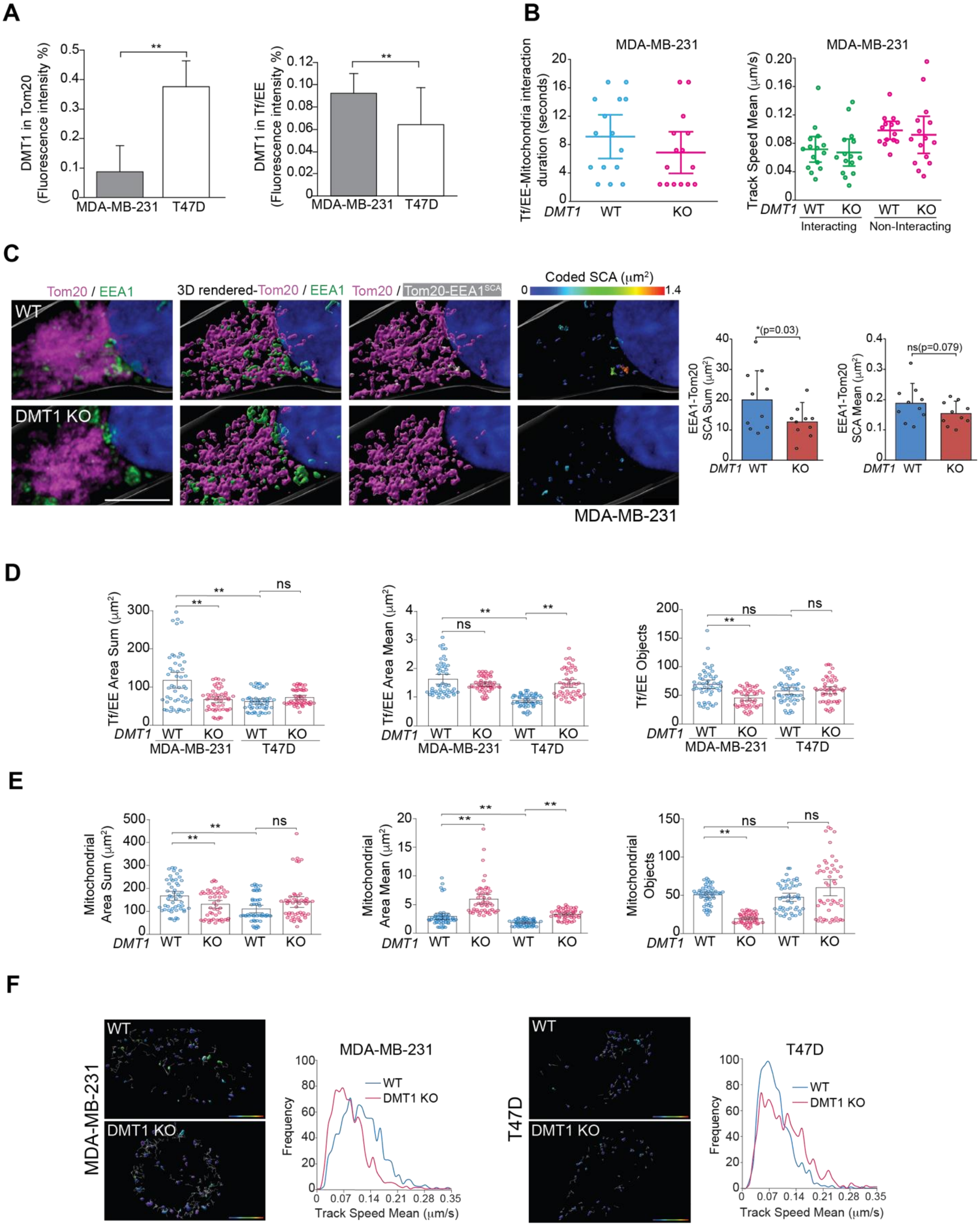

Figure S2

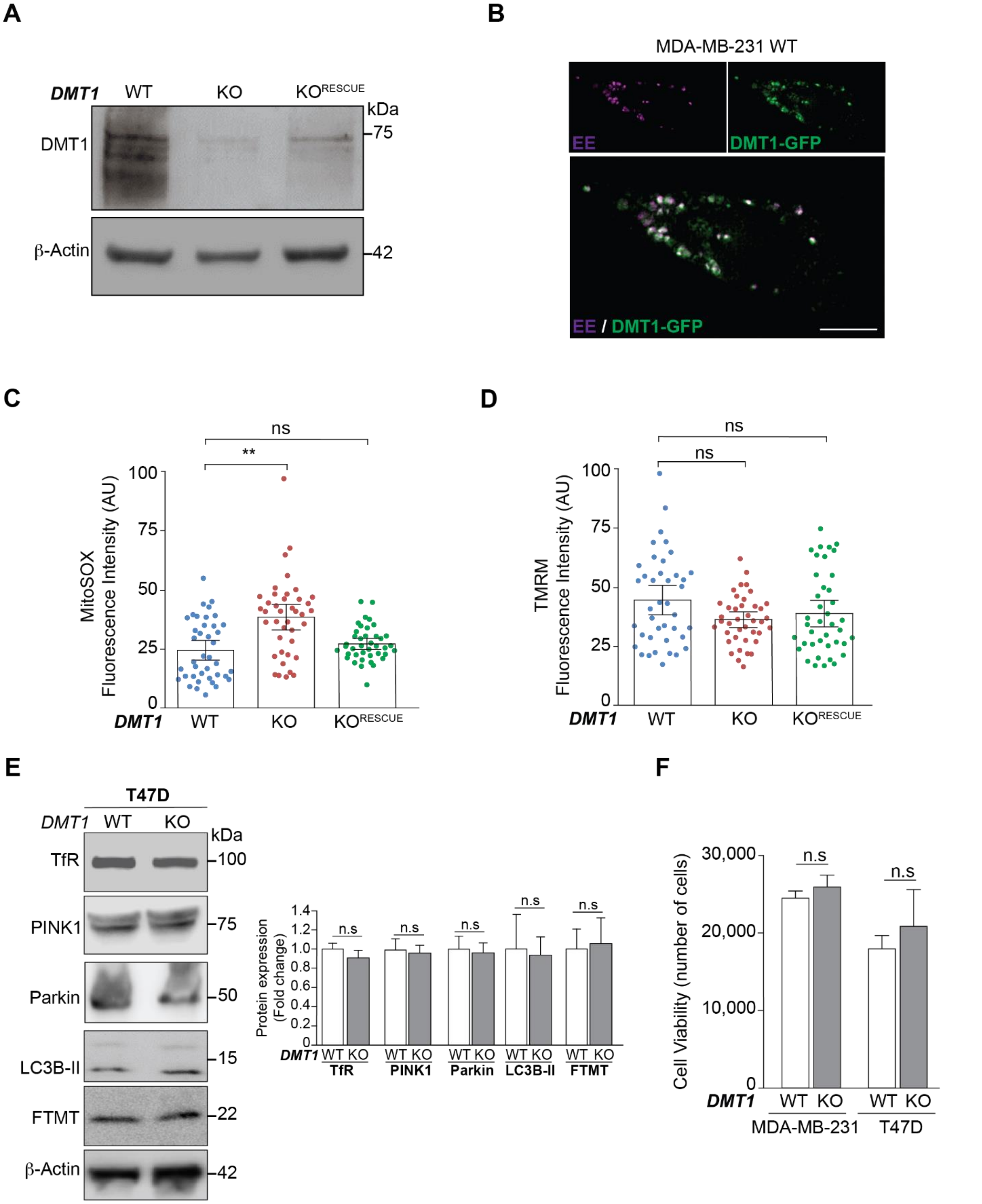

A
DMT1 WT KO KO^RESCUE
kDa
DMT1
75
β-Actin
42
B
MDA-MB-231 WT
EE
DMT1-GFP
EE / DMT1-GFP
C
ns
**
MitoSOX Fluorescence Intensity (AU)
100
75
50
25
0
DMT1 WT KO KO^RESCUE
D
ns
ns
TMRM Fluorescence Intensity (AU)
100
75
50
25
0
DMT1 WT KO KO^RESCUE
E
T47D
DMT1 WT KO
kDa
TfR
100
PINK1
75
Parkin
50
LC3B-II
15
FTMT
22
β-Actin
42
Protein expression (Fold change)
1.4
1.2
1.0
0.8
0.6
0.4
0.2
0
n.s
DMT1 WT KO WT KO WT KO WT KO WT KO
TfR PINK1 Parkin LC3B-II FTMT
F
Cell Viability (number of cells)
30,000
20,000
10,000
0
n.s
DMT1 WT KO WT KO
MDA-MB-231 T47D

Figure S3

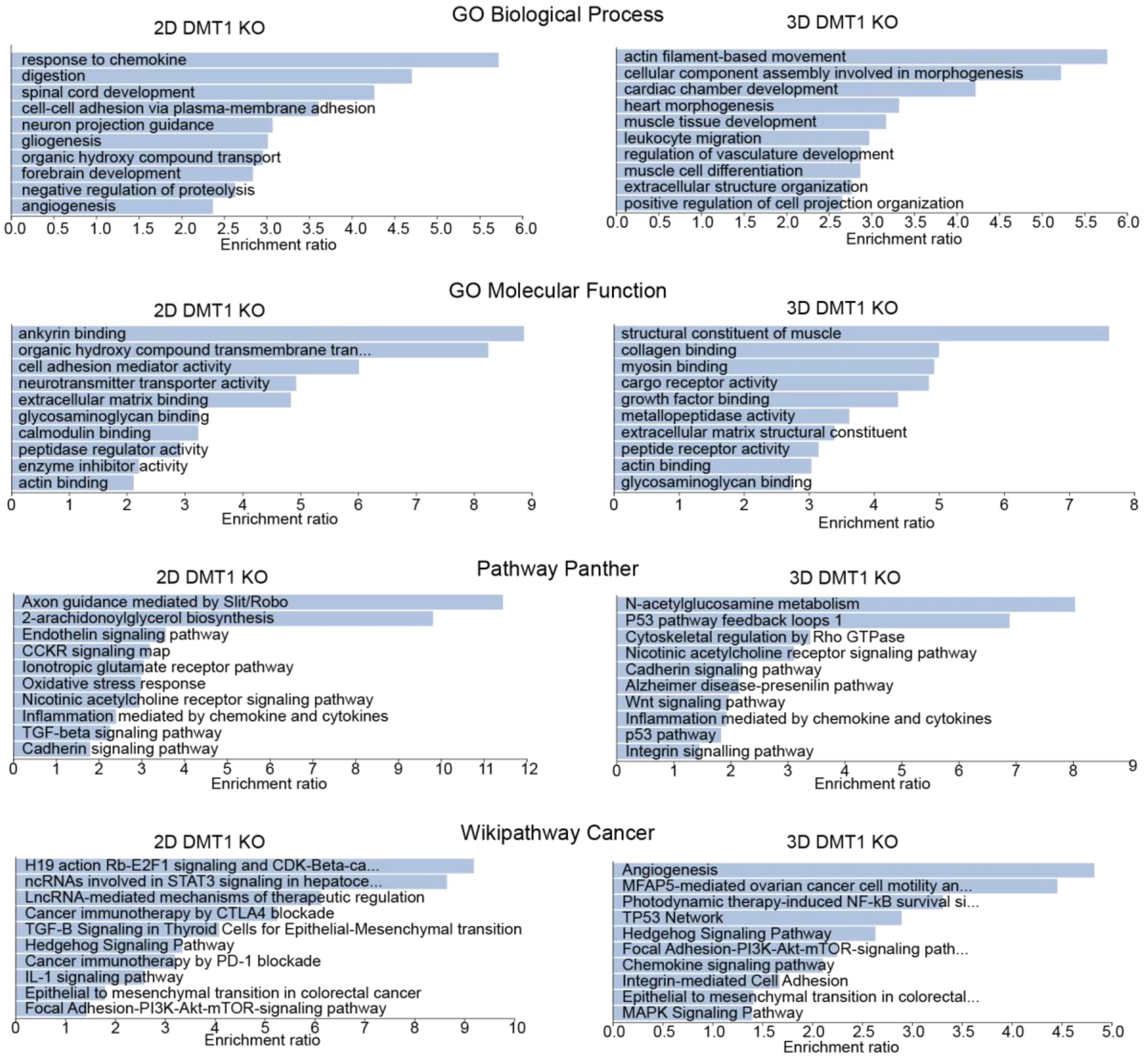

GO Biological Process
2D DMT1 KO
response to chemokine
digestion
spinal cord development
cell-cell adhesion via plasma-membrane adhesion
neuron projection guidance
gliogenesis
organic hydroxy compound transport
forebrain development
negative regulation of proteolysis
angiogenesis
0.0 0.5 1.0 1.5 2.0 2.5 3.0 3.5 4.0 4.5 5.0 5.5 6.0
Enrichment ratio
3D DMT1 KO
actin filament-based movement
cellular component assembly involved in morphogenesis
cardiac chamber development
heart morphogenesis
muscle tissue development
leukocyte migration
regulation of vasculature development
muscle cell differentiation
extracellular structure organization
positive regulation of cell projection organization
0.0 0.5 1.0 1.5 2.0 2.5 3.0 3.5 4.0 4.5 5.0 5.5 6.0
Enrichment ratio
GO Molecular Function
2D DMT1 KO
ankyrin binding
organic hydroxy compound transmembrane tran...
cell adhesion mediator activity
neurotransmitter transporter activity
extracellular matrix binding
glycosaminoglycan binding
calmodulin binding
peptidase regulator activity
enzyme inhibitor activity
actin binding
0 1 2 3 4 5 6 7 8 9
Enrichment ratio
3D DMT1 KO
structural constituent of muscle
collagen binding
myosin binding
cargo receptor activity
growth factor binding
metallopeptidase activity
extracellular matrix structural constituent
peptide receptor activity
actin binding
glycosaminoglycan binding
0 1 2 3 4 5 6 7 8
Enrichment ratio
Pathway Panther
2D DMT1 KO
Axon guidance mediated by Slit/Robo
2-arachidonoylglycerol biosynthesis
Endothelin signaling pathway
CCKR signaling map
Ionotropic glutamate receptor pathway
Oxidative stress response
Nicotinic acetylcholine receptor signaling pathway
Inflammation mediated by chemokine and cytokines
TGF-beta signaling pathway
Cadherin signaling pathway
0 1 2 3 4 5 6 7 8 9 10 11 12
Enrichment ratio
3D DMT1 KO
N-acetylglucosamine metabolism
P53 pathway feedback loops 1
Cytoskeletal regulation by Rho GTPase
Nicotinic acetylcholine receptor signaling pathway
Cadherin signaling pathway
Alzheimer disease-presenilin pathway
Wnt signaling pathway
Inflammation mediated by chemokine and cytokines
p53 pathway
Integrin signalling pathway
0 1 2 3 4 5 6 7 8 9
Enrichment ratio
Wikipathway Cancer
2D DMT1 KO
H19 action Rb-E2F1 signaling and CDK-Beta-ca...
ncRNAs involved in STAT3 signaling in hepatoce...
LncRNA-mediated mechanisms of therapeutic regulation
Cancer immunotherapy by CTLA4 blockade
TGF-B Signaling in Thyroid Cells for Epithelial-Mesenchymal transition
Hedgehog Signaling Pathway
Cancer immunotherapy by PD-1 blockade
IL-1 signaling pathway
Epithelial to mesenchymal transition in colorectal cancer
Focal Adhesion-PI3K-Akt-mTOR-signaling pathway
0 1 2 3 4 5 6 7 8 9 10
Enrichment ratio
3D DMT1 KO
Angiogenesis
MFAP5-mediated ovarian cancer cell motility an...
Photodynamic therapy-induced NF-kB survival si...
TP53 Network
Hedgehog Signaling Pathway
Focal Adhesion-PI3K-Akt-mTOR-signaling path...
Chemokine signaling pathway
Integrin-mediated Cell Adhesion
Epithelial to mesenchymal transition in colorectal...
MAPK Signaling Pathway
0.0 0.5 1.0 1.5 2.0 2.5 3.0 3.5 4.0 4.5 5.0
Enrichment ratio

Figure S4

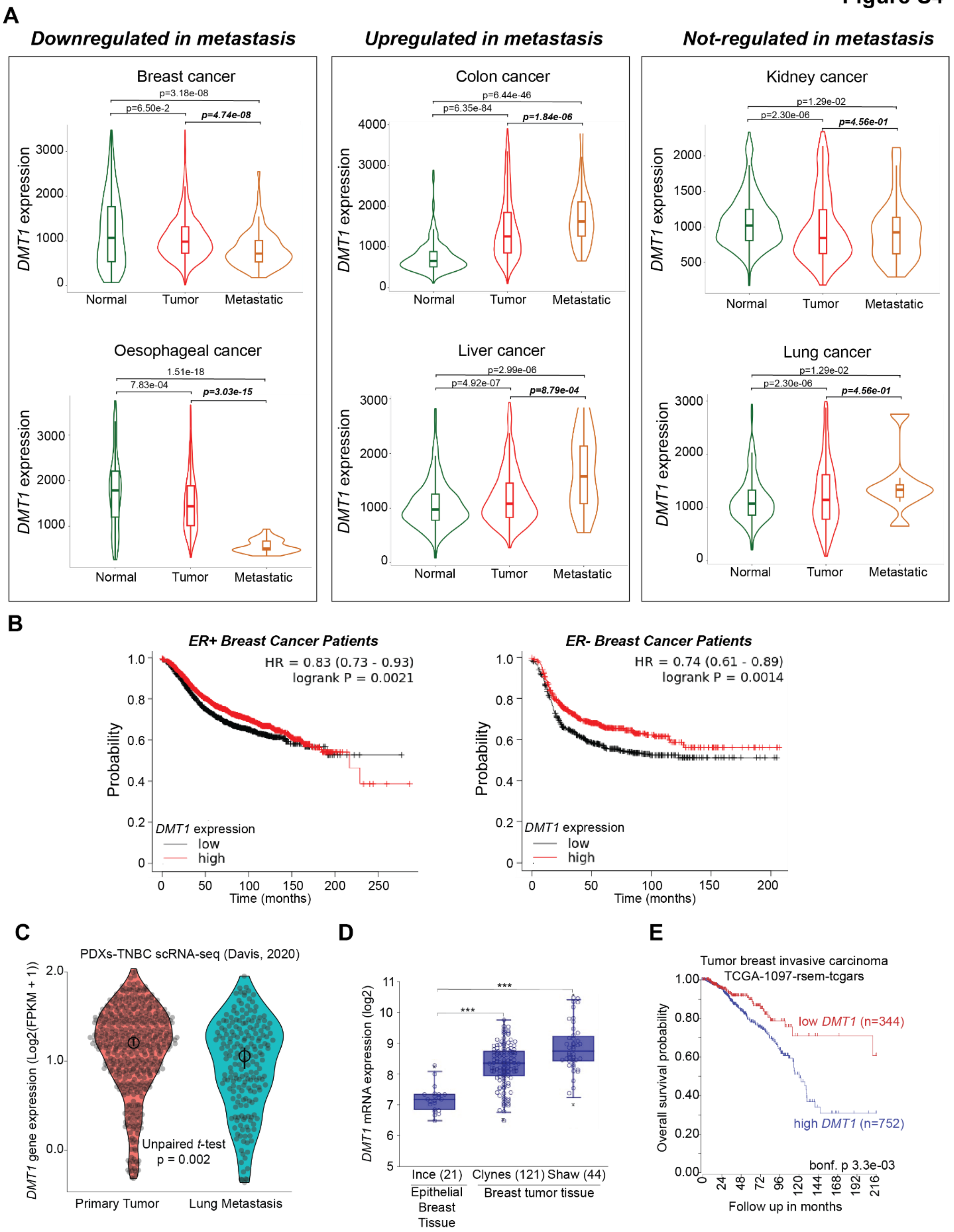

A
Downregulated in metastasis
Upregulated in metastasis
Not-regulated in metastasis
Breast cancer
p=3.18e-08
p=6.50e-2
p=4.74e-08
DMT1 expression
Normal
Tumor
Metastatic
Oesophageal cancer
1.51e-18
7.83e-04
p=3.03e-15
Colon cancer
p=6.44e-46
p=6.35e-84
p=1.84e-06
Liver cancer
p=2.99e-06
p=4.92e-07
p=8.79e-04
Kidney cancer
p=1.29e-02
p=2.30e-06
p=4.56e-01
Lung cancer
p=1.29e-02
p=2.30e-06
p=4.56e-01
B
ER+ Breast Cancer Patients
HR = 0.83 (0.73 - 0.93)
logrank P = 0.0021
ER- Breast Cancer Patients
HR = 0.74 (0.61 - 0.89)
logrank P = 0.0014
Probability
DMT1 expression
low
high
Time (months)
C
PDXs-TNBC scRNA-seq (Davis, 2020)
DMT1 gene expression (Log2(FPKM + 1))
Unpaired t-test
p = 0.002
Primary Tumor
Lung Metastasis
D
DMT1 mRNA expression (log2)
***
***
Ince (21)
Clynes (121)
Shaw (44)
Epithelial Breast Tissue
Breast tumor tissue
E
Tumor breast invasive carcinoma
TCGA-1097-rsem-tcgars
Overall survival probability
low DMT1 (n=344)
high DMT1 (n=752)
bonf. p 3.3e-03
Follow up in months

Supplementary Table S1		
**RESOURCES**	**SOURCE**	**IDENTIFIER**
**Chemicals, peptides and recombinant proteins**		
Protease inhibitor cocktail	Roche	Cat# 04693116001
4X Laemmli sample buffer	BioRad	Cat# 1610747
4-20% Mini-Protean TGX gels	BioRad	Cat# 4561094
Bovine Serum Albumin	Millipore-Sigma	Cat# A9647
2-b-Mercaptoethanol	Millipore-Sigma	Cat# M3148
Clarity Max Western ECL	BioRad	Cat# 1705062
DMEM high glucose, no glutamine, no phenol red	Thermo Fisher Scientific	Cat# 31053-028
Fetal Bovine serum (FBS)	ATCC	Cat# 30-2020
Fish gelatin solution (FGS)	Millipore-Sigma	Cat# G7765
DAPI	Thermo Fisher Scientific	Cat# D1306
Mitotracker Red FM	Thermo Fisher Scientific	Cat# M22425
RDA		
O_2-sensitive MMIR1 nanoparticle probe		
FerroOrange	Dojindo	Cat# F374
MitoSOX Red	Thermo Fisher Scientific	Cat# M36008

TMRM	Thermo Fisher Scientific	Cat# T668
Transferrin Alexa Fluor-488	Thermo Fisher Scientific	Cat# T13342
Transferrin Alexa Fluor-647	Thermo Fisher Scientific	Cat# T23366
Sodium Piruvate (100 mM)	Thermo Fisher Scientific	Cat# 11360070
D-(+)-Glucose solution	Millipore-Sigma	Cat# G8769
Matrigel® Matrix	BD Biosciences	Cat# 356237
Puromycin Dihydrochloride	Thermo Fisher Scientific	Cat# A1113803
Geneticin (G418 Sulfate)	Thermo Fisher Scientific	Cat# 11811023
TransIT®-2020 Transfection Reagent	Mirus	Cat# MIR 5400
X-tremeGENE™ HP DNA Transfection Reagent	Millipore-Sigma	Cat# XTGHP-RO
Polybrene Infection/Transfection Reagent	Millipore-Sigma	Cat# TR-1003-G
Hygromycin B	Thermo Fisher Scientific	Cat# 10687010
**Antibodies**		
Mouse anti-SLC11A2 (DMT1)	Abcam	Cat# ab55735
Rabbit anti-TOM20	Abcam	Cat# ab186735
Mouse anti-TOM20	SCBT	Cat# SC-17764
Rabbit anti-PMPCB	Proteintech	Cat# 16064-1-AP
Mouse anti-Transferrin Receptor	Abcam	Cat# ab1086
Rabbit anti-Ferroportin (SLC40A1)	Novusbio	Cat# NBP1-21502SS
Rabbit anti-Ferritin (FTH)	Cell Signaling	Cat# 3998
Rabbit anti-PINK1	Cell Signaling	Cat# 6946S
Mouse anti-Parkin	Cell Signaling	Cat# 4211S
Rabbit anti-mitochondrial ferritin	Abcam	Cat# ab124889
Rabbit anti-LC3B	Cell Signaling	Cat# 3868S

Mouse anti-beta-Actin-Peroxidase	Millipore-Sigma	Cat# A3854
Mouse anti-EEA1	BD Biosciences	Cat# 610456
Donkey anti-mouse Alexa Fluor 488	Abcam	Cat# ab181289
Donkey anti-rabbit Alexa Fluor 568	Abcam	Cat# ab175694
Rabbit HRP-conjugated IgG	Cell Signaling	Cat# 7074S
Mouse HRP-conjugated IgG	Cell Signaling	Cat# 7076S
**Experimental models: Cell lines**		
MDA-MB-231	ATCC	Cat# HTB-26
T47D	ATCC	Cat# HTB-133
E0771-GFP	Dr. Bravo-Cordero Lab	N/A
HEK293T	ATCC	Cat# CRL-11268
MDA-MB-231-TGL-Parental	Dr. Massague Lab	N/A
MDA-MB-231-TGL-BrM2	Dr. Massague Lab	N/A
MDA-MB-231-TGL-LM2	Dr. Massague Lab	N/A
**Oligonucleotides**		
sgRNA sequence targeting *DMT1* (*SLC11A2*) 5'-TGAGAAGATCTCCATTCCTG-3'	Dr. Fen Zhang Lab (Broad Institute)	N/A
**Recombinant DNA**		
pLentiCRISPRv2-DMT1	GenScript	Item ID# U324RFE290-2
psPAX2	Addgene	Cat# 12260
pCMV-VSV-G	Addgene	Cat# 8454
pEGFP-C1-mouseDMT1 isoform 2	Dr. Jerry Kaplan Lab	N/A
**Experimental models: Organisms/Strains**		
Female, five-weeks-old NSG™ mice NOD.Cg-Prkdc<scid>Il2rg<tm1Wjl>SzJ	Jackson Laboratories	stock# 005557
**Deposited data**		
RNA sequencing data	This paper	GEO: GSE226059

scRNAseq from triple-negative breast cancer (PDXs) primary tumors and their matching lung micrometastases	Reference [68]	GEO: GSE123837
**Software and algorithms**		
Imaris software	Oxford Instruments	https://imaris.oxinst.com/
FIJI	NIH	https://imagej.net/software/fiji/
Graphpad Prism 6	Prism	https://www.graphpad.com
DESeq2 v 1.30.0	Bioconductor	https://bioconductor.org/packages/release/bioc/html/DESeq2.html
R package	The R Project for Statistical Computing	https://www.r-project.org/
LASX Software	Leica	https://www.leica-microsystems.com/products/microscope-software/p/leica-las-x-ls
