# Supplementary material for: DMT1-dependent endosome-mitochondria interactions regulate mitochondrial iron translocation and metastatic outgrowth
Source: Oncogene. 2024 Jan 6;43(9):650–67. doi: 10.1038/s41388-023-02933-x (PMC10890933; doi:10.1038/s41388-023-02933-x)
Supplement: Supplementary file 1 [file 41388_2023_2933_MOESM5_ESM.docx]

**Supplementary Information**

**Figure Supplementary S1.** 

**(A)** Fluorescence intensity levels between EE and DMT1 in T47D and MDA-MB-231 breast cancer cells. Fluorescent data from the IF experiments shown in **Figure 1A** was analyzed. Bar graphs show the fluorescent intensity of both DMT1 in Tom20 and DMT1 in EE. Unpaired *t*-test; **p<0.05. 

**(B)** The duration of EE-mitochondria interaction was analyzed in a subset of 15 endosomes in both parental WT and DMT1 KO MDA-MB-231 cells, as well as the Track speed means of subsets of interacting versus non-interacting EE was compared.

**(C)** Fluorescent images were 3D rendered and analyzed using IMARIS 9.6 software to quantify EE-Tom20 SCA sum and EE-Tom20 SCA mean (n=10 cells). Unpaired *t*-test.

(**D**) EE area sum, EE area mean, and the number of EE objects are shown in indicated cell lines.

**(E)** Mitochondrial area sum, mitochondrial area means, and the number of mitochondrial objects is shown in indicated cell lines. Parameters were calculated using data from 10 individual cells in 5 consecutive time intervals per condition and analyzed separately (n=50). One-way ANOVA with Bonferroni post-hoc test. **p<0.01. ns: non-significant (p>0.05).

**(F)** Representative images show color-coded EE Track Speed Mean in MDA-MB-231 and T47D WT and DMT1 KO cells. Endosomal track length and trajectories are depicted. MDA-MB-231 endosomal track speed mean histogram shows a higher frequency of slower EE in DMT1 KO compared to WT parental cells. T47D EE track speed mean histogram shows a similar EE track speed mean frequency between WT and DMT1 KO cells. Scale bar = 10 μm.

**Figure Supplementary S2.** 

**(A)** Representative immunoblots of DMT1 in MDA-MB-231 WT, DMT1 KO, and DMT1 KO^RESCUE^ cells. β-Actin was used as a loading control. 

(**B**) EE colocalizes with DMT1-GFP. MDA-MB-231 cells were transiently transfected with pDMT1-GFP and then incubated with Tf-AlexaFluor 647 for 2 min for EE labeling. Live cells were imaged using a Leica Thunder microscope (63x oil immersion objective). Images demonstrate the co-distribution of both markers. Scale bar = 10 μm.

**(C-D)** Normalized fluorescence quantification of MitoSOX (C) and TMRM (D) dyes in indicated cells at steady state. Graphs show the quantification of fluorescence intensity in arbitrary units (AU). Fluorescence intensity was analyzed using ImageJ software in 40 cells per condition. One-way ANOVA with Bonferroni post-hoc test. **p<0.01. ns: non-significant (p>0.05).

**(E)** In T47D breast cancer cells DMT1 silencing did not alter the protein expression levels of TfR or mitophagy-related markers. (A) Immunoblots and (B) normalized densitometry quantification of TfR, PINK1, Parkin, LC3B-II, and mitochondrial ferritin (FTMT) in T47D WT and T47D DMT1 KO cells. β-Actin was used as a loading control. Unpaired *t*-test; n = 3; ns = not significant.

**(F)** DMT1 silencing did not affect cell viability in either MDA-MB-231 or T47D breast cancer cells. 3 000 cells were plated in 96-well plates (8-12 wells per condition) and incubated for 3 days at 37°C in 5% CO_2_. Cells were fixed with 4% PFA, permeabilized with 0.1 % Triton, and then stained with DAPI (10 μg/ml) for 10 minutes. The number of cells was counted using Cytation 5 (Biotek) according to manufacturer instructions. Unpaired *t*-test; n = 3; ns = not significant.

**Figure Supplementary S3.** 

Additional Gene Ontology analyses from RNA seq data upon DMT1 KO in MDA-MB-231 cells 2D and 3D cell culture conditions. Functional enrichment analysis was performed using WebGestalt (WEB-based Gene Set Analysis Toolkit, [http://www.webgestalt.org](http://www.webgestalt.org)) [39].

**Figure Supplementary S4.** 

**(A)** Violin plots of DMT1 gene expression in cancers where its expression is downregulated in metastasis i.e., breast, oesophageal, and prostate cancer (left panel), upregulated in metastasis i.e., colon and liver cancer (mid panel) and not regulated in metastatic tissue i.e., kidney and lung cancer. Data was generated using the TNMplot website tool ([www.tnmplot.com](http://www.tnmplot.com)). Normal, tumor, and metastatic tissue gene comparison was statistically analyzed using the Kruskal–Wallis test. The statistical significance cutoff was set at p < 0.01. P-values obtained from a Kruskal–Wallis post-hoc Dunn’s test are indicated in each graph.

**(B)** Kaplan- Meier survival plots obtained from [www.KMplot.com](http://www.KMplot.com) breast cancer database of both ER+ and ER- breast cancer patients (left and right panel, respectively). Hazard ratio (HR) and log-rank P values are indicated.  

**(C)** DMT1 levels in single cells from patient-derived xenografts (PDXs) primary tumors and their matching lung micrometastases (GSE123837).

**(D)** 21 samples of epithelial breast tissue were compared with two breast tumor cohorts (Clynes and Shaw, 121 and 42 samples, respectively) using the R2 genomics analysis and visualization platform.

**(E)** High DMT1 (SLC11A2) mRNA expression is associated with decreased overall survival probability in tumor breast invasive carcinoma patients. Kaplan Meier's overall survival probability analysis of a tumor breast invasive carcinoma cohort is shown. The long-rank test result is indicated. The cut-off was calculated using the scanning method.

**Supplementary Figures**

| **Supplementary Table S1** |  |  |  |
| --- | --- | --- | --- |
| **RESOURCES** | **SOURCE** | **IDENTIFIER** |  |
| **Chemicals, peptides and recombinant proteins** |  |  |  |
| Protease inhibitor cocktail | Roche | Cat# 04693116001 |  |
| 4X Laemmli sample buffer | BioRad | Cat# 1610747 |  |
| 4-20% Mini-Protean TGX gels | BioRad | Cat# 4561094 |  |
| Bovine Serum Albumin | Millipore-Sigma | Cat# A9647 |  |
| 2-b-Mercaptoethanol | Millipore-Sigma | Cat# M3148 |  |
| Clarity Max Western ECL | BioRad | Cat# 1705062 |  |
| DMEM high glucose, no glutamine, no phenol red | Thermo Fisher Scientific | Cat# 31053-028 |  |
| Fetal Bovine serum (FBS) | ATCC | Cat# 30-2020 |  |
| Fish gelatin solution (FGS) | Millipore-Sigma | Cat# G7765 |  |
| DAPI | Thermo Fisher Scientific | Cat# D1306 |  |
| Mitotracker Red FM | Thermo Fisher Scientific | Cat# M22425 |  |
| RDA |  |  |  |
| O_2_-sensitive MMIR1 nanoparticle probe |  |  |  |
| FerroOrange | Dojindo | Cat# F374 |  |
| MitoSOX Red | Thermo Fisher Scientific | Cat# M36008 |  |
| TMRM | Thermo Fisher Scientific | Cat# T668 |  |
| Transferrin Alexa Fluor-488 | Thermo Fisher Scientific | Cat# T13342 |  |
| Transferrin Alexa Fluor-647 | Thermo Fisher Scientific | Cat# T23366 |  |
| Sodium Piruvate (100 mM) | Thermo Fisher Scientific | Cat# 11360070 |  |
| D-(+)-Glucose solution | Millipore-Sigma | Cat# G8769 |  |
| Matrigel® Matrix | BD Biosciences | Cat# 356237 |  |
| Puromycin Dihydrochloride | Thermo Fisher Scientific | Cat# A1113803 |  |
| Geneticin (G418 Sulfate) | Thermo Fisher Scientific | Cat# 11811023 |  |
| TransIT®-2020 Transfection Reagent | Mirus | Cat# MIR 5400 |  |
| X-tremeGENE™ HP DNA Transfection Reagent | Millipore-Sigma | Cat# XTGHP-RO |  |
| Polybrene Infection/Transfection Reagent | Millipore-Sigma | Cat# TR-1003-G |  |
| Hygromycin B | Thermo Fisher Scientific | Cat# 10687010 |  |
| **Antibodies** |  |  |  |
| Mouse anti-SLC11A2 (DMT1) | Abcam | Cat# ab55735 |  |
| Rabbit anti-TOM20 | Abcam | Cat# ab186735 |  |
| Mouse anti-TOM20 | SCBT | Cat# SC-17764 |  |
| Rabbit anti-PMPCB | Proteintech | Cat# 16064-1-AP |  |
| Mouse anti-Transferrin Receptor | Abcam | Cat# ab1086 |  |
| Rabbit anti-Ferroportin (SLC40A1) | Novusbio | Cat# NBP1-21502SS |  |
| Rabbit anti-Ferritin (FTH) | Cell Signaling | Cat# 3998 |  |
| Rabbit anti-PINK1 | Cell Signaling | Cat# 6946S |  |
| Mouse anti-Parkin | Cell Signaling | Cat# 4211S |  |
| Rabbit anti-mitochondrial ferritin | Abcam | Cat# ab124889 |  |
| Rabbit anti-LC3B | Cell Signaling | Cat# 3868S |  |
| Mouse anti-beta-Actin-Peroxidase | Millipore-Sigma | Cat# A3854 |  |
| Mouse anti-EEA1 | BD Biosciences | Cat# 610456 |  |
| Donkey anti-mouse Alexa Fluor 488 | Abcam | Cat# ab181289 |  |
| Donkey anti-rabbit Alexa Fluor 568 | Abcam | Cat# ab175694 |  |
| Rabbit HRP-conjugated IgG | Cell Signaling | Cat# 7074S |  |
| Mouse HRP-conjugated IgG | Cell Signaling | Cat# 7076S |  |
| **Experimental models: Cell lines** |  |  |  |
| MDA-MB-231 | ATCC | Cat# HTB-26 |  |
| T47D | ATCC | Cat# HTB-133 |  |
| E0771-GFP | Dr. Bravo-Cordero Lab | N/A |  |
| HEK293T | ATCC | Cat# CRL-11268 |  |
| MDA-MB-231-TGL-Parental | Dr. Massague Lab | N/A |  |
| MDA-MB-231-TGL-BrM2 | Dr. Massague Lab | N/A |  |
| MDA-MB-231-TGL-LM2 | Dr. Massague Lab | N/A |  |
| **Oligonucleotides** |  |  |  |
| sgRNA sequence targeting *DMT1* (*SLC11A2*) 5’-TGAGAAGATCTCCATTCCTG-3’ | Dr. Fen Zhang Lab (Broad Institute) | N/A |  |
| **Recombinant DNA** |  |  |  |
| pLentiCRISPRv2-DMT1 | GenScript | Item ID# U324RFE290-2 |  |
| psPAX2 | Addgene | Cat# 12260 |  |
| pCMV-VSV-G | Addgene | Cat# 8454 |  |
| pEGFP-C1-mouseDMT1 isoform 2 | Dr. Jerry Kaplan Lab | N/A |  |
| **Experimental models: Organisms/Strains** |  |  |  |
| Female, five-weeks-old NSG™ mice NOD.Cg-Prkdc<scid>II2rg<tm1Wjl>SzJ | Jackson Laboratories | stock# 005557 |  |
| **Deposited data** |  |  |  |
| RNA sequencing data | This paper | GEO: GSE226059 |  |
| scRNAseq from triple-negative breast cancer (PDXs) primary tumors and their matching lung micrometastases | Reference [68] | GEO: GSE123837 |  |
| **Software and algorithms** |  |  |  |
| Imaris software | Oxford Instruments | [https://imaris.oxinst.com/ ](https://imaris.oxinst.com/) |  |
| FIJI | NIH | [https://imagej.net/software/fiji/](https://imagej.net/software/fiji/) |  |
| Graphpad Prism 6 | Prism | [https://www.graphpad.com ](https://www.graphpad.com/) |  |
| DESeq2 v 1.30.0 | Bioconductor | https://bioconductor.org/packages/release/bioc/html/DESeq2.html |  |
|  |  |  |  |
| R package | The R Project for Statistical Computing | [https://www.r-project.org/ ](https://www.r-project.org/) |  |
| LASX Software | Leica | [https://www.leica-microsystems.com/products/microscope-software/p/leica-las-x-ls ](https://www.leica-microsystems.com/products/microscope-software/p/leica-las-x-ls) |  |
